# Introducing activation functions into segmented regression model to address lag effects of interventions

**DOI:** 10.1186/s12874-023-02098-x

**Published:** 2023-11-24

**Authors:** Xiangliang Zhang, Kunpeng Wu, Yan Pan, Wenfang Zhong, Yixiang Zhou, Tingting Guo, Rong Yin, Wen Chen

**Affiliations:** 1 Department of Medical Statistics, School of Public Health, Sun Yat-sen University, Guangzhou, China; 2 Center for Migrant Health Policy, Sun Yat-sen University, Guangzhou, China

**Keywords:** Intervention evaluation, Interrupted time series, Activation functions, Segmented regression, Statistical methods, Simulation study

## Abstract

The interrupted time series (ITS) design is widely used to examine the effects of large-scale public health interventions and has the highest level of evidence validity. However, there is a notable gap regarding methods that account for lag effects of interventions.

To address this, we introduced activation functions (ReLU and Sigmoid) to into the classic segmented regression (CSR) of the ITS design during the lag period. This led to the proposal of proposed an optimized segmented regression (OSR), namely, OSR-ReLU and OSR-Sig. To compare the performance of the models, we simulated data under multiple scenarios, including positive or negative impacts of interventions, linear or nonlinear lag patterns, different lag lengths, and different fluctuation degrees of the outcome time series. Based on the simulated data, we examined the bias, mean relative error (MRE), mean square error (MSE), mean width of the 95% confidence interval (CI), and coverage rate of the 95% CI for the long-term impact estimates of interventions among different models.

OSR-ReLU and OSR-Sig yielded approximately unbiased estimates of the long-term impacts across all scenarios, whereas CSR did not. In terms of accuracy, OSR-ReLU and OSR-Sig outperformed CSR, exhibiting lower values in MRE and MSE. With increasing lag length, the optimized models provided robust estimates of long-term impacts. Regarding precision, OSR-ReLU and OSR-Sig surpassed CSR, demonstrating narrower mean widths of 95% CI and higher coverage rates.

Our optimized models are powerful tools, as they can model the lag effects of interventions and provide more accurate and precise estimates of the long-term impact of interventions. The introduction of an activation function provides new ideas for improving of the CSR model.

**Supplementary Information:**

The online version contains supplementary material available at 10.1186/s12874-023-02098-x.

## Introduction

The interrupted time series (ITS) design developed by Box and Tiao in 1975, [1] stands as a quasi-experimental design with the strongest evidence validity [2, 3]. It is possibly the most practical method for examining the effects of large-scale public health policies given ethical, social, or logistical constraints [4]. It involves collecting data both before and after the intervention, and analyzing the time series data to determine whether the intervention has led to a significant change in the outcome beyond the expected temporal trends. Segmented regression, the most classic and widely used statistical method for evaluating the effects of interventions under an ITS design with the aggregated time series data (weekly, monthly, etc.), [5, 6] is a powerful method that accounts for underlying trends and has a high capacity to infer causation [7]. Classic segmented regression (CSR) sets the interruption a priori at a fixed point in the outcome time series, typically the nominal intervention time point, to distinguish between the pre- and post- intervention phases [8]. In the pre- and post-intervention phases, the CSR model typically requires data to be linear, homoscedastic, independent, normal, and stationary [3, 9, 10]. After the fixed interruption point (initial post-intervention phase), the linear data requirement of the CSR model makes the impacts of interventions perceived as immediate, direct, and leapfrogged at a fixed point, which may be violated in intervention evaluations [6].

However, some interventions and exposures in practice may have gradual or delayed impacts on the outcome time series, as reported in many studies [11–14]. For example, the nursing intervention carried out by the Oncology Advanced Practice Nurse in the USA had a lag effect on the quality of life of post-surgical women with gynecologic cancer, [11] and the effects of the Medicare Act on hospital admission tended to emerge gradually after its adoption [12]. In addition, there is substantial evidence that air pollution exposure has a lag effect on respiratory and cardiovascular disease-related mortality and morbidity [13–16]. The effect of supra-threshold heat exposure on mortality persisted for several days, and the cumulative effect remained high even after 30 days of exposure in Seoul and Incheon [17].

In the above-mentioned situations, it takes time for interventions to reach the long-term trajectory of their effectiveness. The time frame required for an intervention to reach its long-term trajectory is referred to as the lag period, and the length of this time frame is the lag length. During the lag period, the effect of the intervention was gradually applied to the outcome time series. We defined this gradual or delayed effect of the intervention as the “lag effect”. The CSR model either ignores the time points of the lag period and models the entire time series directly, or removes them, and then models the remaining time points [5, 18]. Direct modeling using the CSR model may violate the basic linearity requirement for the regression model within the post-intervention phases, and the parameter estimates can be biased and inconsistent. Censoring (removing time points of the lag period) not only leaves out data but can also distort parameter estimations [19]. In conclusion, the CSR model fails to model the lag effects of interventions and exposure because of its priori setting of interruptions at only one fixed point in the outcome time series. When interventions have linear or nonlinear lag effects, [20, 21] there is still a lack of sufficient solutions for intervention evaluation using an outcome time series alone. To address this issue, we planned to model the lag effects of interventions by introducing ‘bridge functions’ (activation functions) into the CSR during the lag period, and thus proposed an optimized segmented regression (OSR) model. Activation functions are a family of functions with various forms that are sufficiently flexible to describe linear and nonlinear processes [22]. They are suitable to describe the lag effects of interventions and their different forms correspond to different patterns of lag effects. Their common forms include the ReLU, Sigmoid, hyperbolic tangent, and Mish [23]. In this study, we utilized the most commonly used linear ReLU and nonlinear Sigmoid functions as examples for our analysis. We introduced these two activation functions to model the linear and nonlinear lag patterns, and established the OSR-ReLU and OSR-Sig models, respectively.

To compare the performance of OSR with that of CSR, we simulated a sequence of outcome time series with the characteristic that interventions have delayed impacts on outcome time series. We simulated multiple scenarios, including the positive or negative impacts of interventions, linear or nonlinear lag patterns, different lag lengths, and different fluctuation degrees of the outcome time series. We established a set of criteria to evaluate the performances of the different models [24]. Based on the simulated data, the performance of the models was evaluated by examining the bias of estimates and the mean relative error (MRE), mean square error (MSE), mean width of 95% confidence interval (CI), and coverage rate of 95% CI for the long-term impact estimates of interventions. This was to ascertain whether the optimized models (OSR-ReLU and OSR-Sig) were superior to the classic model (CSR).

## Methods

### Classic segmented regression (CSR)

CSR is widely used in intervention evaluations under the ITS design, and models can be used to analyze aggregated (weekly, monthly, etc.) time-series data that meet linear, homoscedastic, independent, normal, and stationary assumptions.

1 $${Y}_{t}={\beta }_{0}+{\beta }_{1}\times { time }+{\beta }_{2}\times { intervention}+{\beta }_{3} \times \text {post-time}+{\varepsilon }_{t}.$$

The matrix expression of Eq. 1 is:

2 $$\varvec{Y}={\varvec{X}}_{\varvec{C}\varvec{S}\varvec{R}}\text{*}\varvec{\beta }+\varvec{\epsilon }; \varvec{\beta }={\left[{\beta }_{0},{\beta }_{1},{\beta }_{2},{\beta }_{3}\right]}^{T}, {\varvec{X}}_{\varvec{C}\varvec{S}\varvec{R}}=\left[\begin{array}{cccc}1& 1& 0& 0\\ \vdots& \vdots& \vdots& \vdots\\ 1& {T}_{0}& 0& 0\\ 1& {T}_{0}+1& 1& 1\\ \vdots& \vdots& \vdots& \vdots\\ 1& {T}_{e}& 1& {T}_{e}-{T}_{0}\end{array}\right].$$

${Y}_{t}$ is the outcome of interest at time point $t$, which can be a weekly or monthly routine data source (e.g., total monthly births in hospitals). From the initial observation point to the last observation point, $time$ is was used as the variable of the time point ($time=t=1, 2, 3,\dots ,{T}_{e}$). ${T}_{e}$ is the total length of the time series and ${T}_{0}$ is the time point at which the intervention is implemented (nominal intervention time). The intervention was executed using a dummy variable, $intervention$. The values before and after the implementation of the intervention are represented as 0 and 1, respectively. The indicator variable, $\text{post-time}$, is used to track the passing of time after the nominal implementation of the intervention. During the post-implementation phase, the value of $\text{post-time}$ was initially set to 1 and increased over time (${ \text{post-time}} =1, 2, 3, \ldots, {T}_{e}-{T}_{0}<{T}_{e}$). ${\epsilon }_{t}$ is the random error term at the time $t$ with constant variance ${\sigma }_{\epsilon }^{2}$ (independent of $t$), i.e., homogeneity of variance for the regression model (Eq. 1).

The baseline trend of the outcome time series before the implementation is represented by ${\beta }_{1}$. The instant impact of the intervention on ${Y}_{t}$ is reflected by the value of ${\beta }_{2}$. The change in the trend of outcome time series (slopes) is represented by ${\beta }_{3}$, which captures the intervention’s long-term impact.

The CSR model assumes that the intervention effect occurs instantaneously at a certain time and cannot handle the intervention’s lag effect. To address this problem, we introduced activation functions into the CSR model to modify the variables ${intervention}$ and $\text{post-time}$, and proposed an optimization model.

### Optimized segmented regression (OSR)

OSR (Eq. 3) is an improvement of CSR. In the optimized model, we modeled the lag period by introducing different activation functions as follows:

3 $${Y}_{t}={\beta }_{0}+{\beta }_{1}\times { time }+{\beta }_{2}\times F\left(t\right)\times { intervention}+{\beta }_{3}\times F\left(t\right)\times \text{post-time}+{\epsilon }_{t}.$$

The piecewise function $F\left(t\right)$ 

4 $$F\left(t\right)=\left\{\begin{array}{ll} f\left(t-{T}_{0}\right)& ,{T}_{0}\le t\le {T}_{0}+L;\\ 1& , \text{else}.\end{array}\right.$$

$L$ is the deemed lag length. For the activation function $f\left(t\right)$, there are several possible forms, which can fit different lag patterns of intervention effects. The actual lag pattern ofintervention effects determines the form used. In this study, we discuss linear (ReLU) and nonlinear (Sigmoid) patterns: (1) linear: $f\left(t\right)=\frac{1}{\text{L}}*t$, and the corresponding model is denoted by OSR-ReLU; (2) nonlinear: $f\left(t\right)=\frac{a}{\text{1+}{e}^{-t}}+b$, subject to $$\left\{\begin{array}{c}\frac{{a}}{{1+}{e}^{-\left(-\frac{{L}}{2}\right)}}+{b}=0,\\ \frac{{a}}{{1+}{e}^{-\frac{{L}}{2}}}+{b}=1,\end{array}\right.$$ and the corresponding model is denoted by OSR-Sig.

**Fig. 1:**
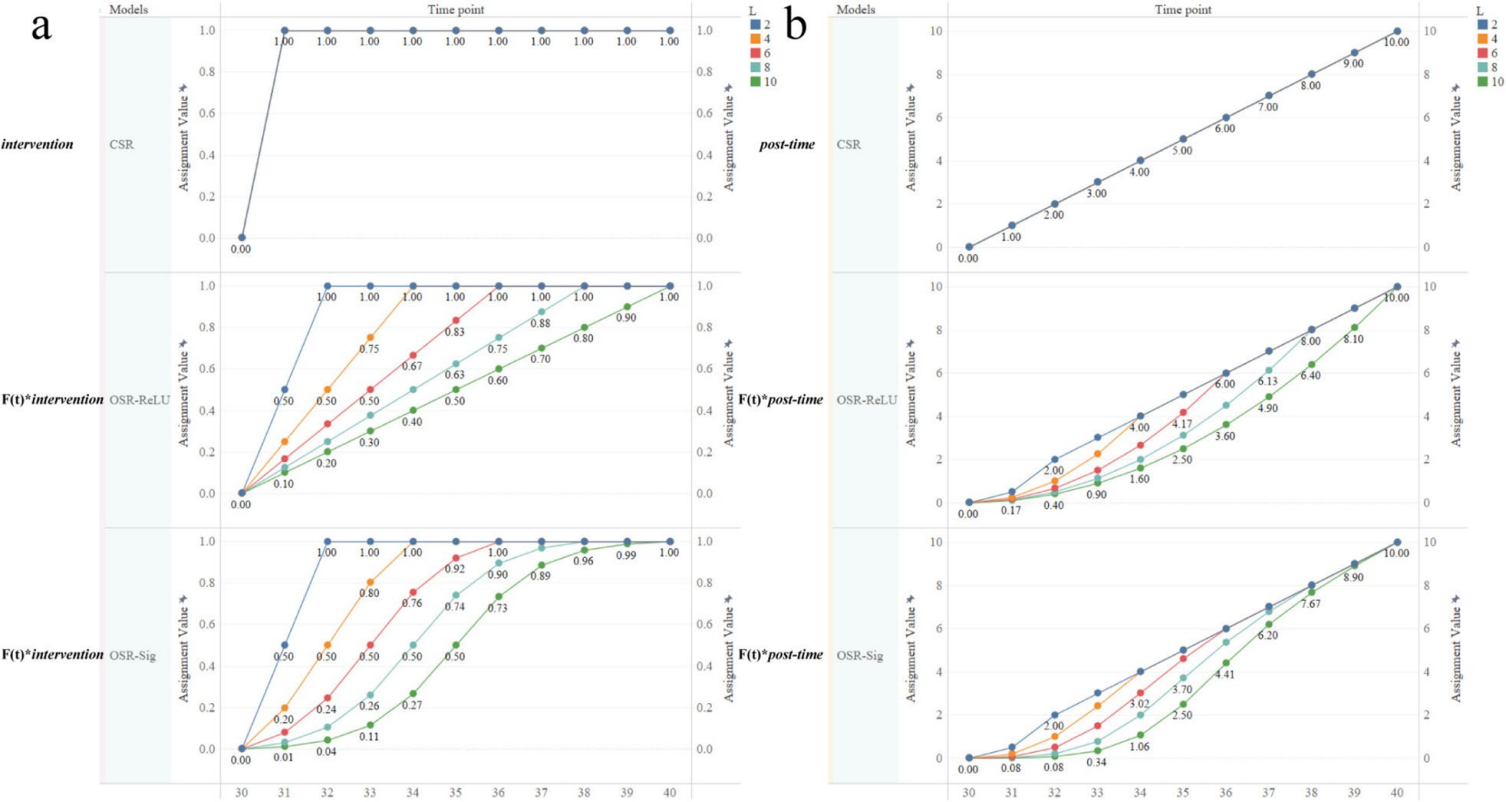
Assignment of ${F}({t})\times \text{ }{intervention}$ and ${F}({t})\times \text{post-time}$ during the lag period for different lag lengths ${L}$. Panels **a** and **b** depict the assignment results of variables $F\left(t\right)\times \text{ }{intervention}$ and $F\left(t\right)\times \text{post-time}$ (for the CSR model, they depict the assignment results of variables ${intervention}$, and $\text{post-time}$) for different lag lengths $L$ (2, 4, 6, 8, 10) during the lag period for the three different models (CSR, OSR-RuLU and OSR-Sig), respectively. Different colors represent different lag lengths $L$. Blue indicates $L$=2, orange indicates $L$=4, red indicates $L$=6, cyan indicates $L$=8, and green indicates $L$=10

The difference between the CSR and OSR models is the introduction of activation functions to modify the variables ${intervention}$ and $\text{post-time}$ during the lag period. Specifically, the differences in the variable assignments $F\left(t\right)\times \text{ }{intervention}$ and $F\left(t\right)\times \text{post-time}$ are shown in Fig. 1 for lag lengths of 2, 4, 6, 8, and 10 during the lag period. When the lag length *L* = 2, the OSR-ReLU and OSR-Sig models are equivalent, as demonstrated in Supplementary Material - Explanation 1.

### Data simulation

We first set the length of the simulated outcome time series to 60-time points and divided the outcome time series into two equal parts, representing the pre-intervention and post-intervention phases.

In this study, the pre-intervention series for outcome variable generation was defined as ${Y}_{pre}\left(t\right)=1*t+1$. For the post-intervention series of the outcome variable, we considered two situations: the intervention had a positive or negative long-term impact (we set its absolute value to 2) on the outcome time series. Thus, the post-intervention outcome time series were ${Y}_{post-p}\left(t\right)=\left(1+2\right)*t+1=3*t+1$ for positive impact (+ 2) and ${Y}_{post-n}\left(t\right)=\left(1-2\right)*t+1=-1*t+1$ for negative impact (-2).

For the possible lag length $L$, we simulated $L$=2, 4, 6, 8, and 10 for the five different lag lengths. Within the lag length, we used linear and S-smooth interpolation to simulate the linear (ReLU) and nonlinear (Sigmoid) lag patterns separately, so that the simulated outcome time series have different lag patterns of interventions. These two interpolations of the outcome time series $Y\left(t\right)$ are expressed as ${IPLT}_{ReLU}\left(t\right)$ (Eq. 5) and ${IPLT}_{Sig}\left(t\right)$ (Eq. 6).

5 $${IPLT}_{ReLU}\left(t\right)={{t}}_{1}+\frac{Y\left({{t}}_{2}\right)-Y\left({{t}}_{1}\right)}{{{t}}_{2}-{{t}}_{1}}\left(t-{{t}}_{1}\right); {{t}}_{1}\le t\le {{t}}_{2}.$$

6 $${IPLT}_{Sig}\left(t\right)={\frac{{{a}}_{s}}{{1+}{e}^{-t}}+{b}_{s}}; \text{subject to} \left\{\begin{array}{c}\frac{{{a}}_{s}}{\text{1+}{e}^{-\left(-\frac{{{t}}_{2}-{{t}}_{1}+2}{2}\right)}}+{{b}}_{s}=Y\left({{t}}_{1}\right),\\ \frac{{{a}}_{s}}{{1+}{e}^{-\frac{{{t}}_{2}-{{t}}_{1}+2}{2}}}+{{b}}_{s}=Y\left({{t}}_{2}\right),\end{array} {{t}}_{1}\le t\le {{t}}_{2}.\right.$$

${{t}}_{1}$, $Y\left({{t}}_{1}\right)$ and ${{t}}_{2}$, $Y\left({{t}}_{2}\right)$ are the values of the interpolation function at the two corresponding interpolation points. In our simulation, ${{t}}_{1}=30$, ${{t}}_{2}=30+L$ and $Y\left({{t}}_{1}\right)={Y}_{pre}\left(30\right)$, $Y\left({{t}}_{2}\right)={Y}_{post-p}\left(30+L\right)$ or ${Y}_{post-n}\left(30+L\right)$.

We then added a white noise series to the outcome time series to simulate random perturbation of the outcome time series by other factors, including white noises, $N\left(0,{3}^{2}\right)$, $N\left(0,{6}^{2}\right)$ and $N\left(0,{9}^{2}\right)$, a total of three different fluctuation levels of white noise.

#### Possible scenarios of outcome time series

During the simulation process, different outcome time series were generated separately using different parameter settings. For the positive (+ 2) or negative (-2) long-term impact of the intervention and two interpolation methods for the lag period (${IPLT}_{ReLU}$ and ${IPLT}_{Sig}$), there were $2\times 2=4$ types of outcome time series, which were represented by ‘*2&ReLU, 2&Sig, N2&ReLU*, and *N2&Sig’* in this study. Additionally considering five different lag lengths ($L=2, 4, 6, 8, 10$) and three different $\sigma$ of white noises ($N\left(0,{3}^{2}\right)$, $N\left(0,{6}^{2}\right)$ and $N\left(0,{9}^{2}\right)$), the number of possible scenarios for outcome time series was 60 ($2\times 2\times 5\times 3=60$). For one specific simulation scenario, we used $n=\text{1,000}$ simulation repetitions. The outcome time series generated under different simulation scenarios are shown in Supplementary Material Fig S1.

#### Software and execution

The statistical software MATLAB (version 9.6.0.10727) was used to generate the simulated data and model calculations [25–27]. The function ‘*Normrnd*’ in MATLAB was used for the generation of random numbers. For reproducibility, a seed was set at the beginning of the simulation. Tableau Desktop 20021.3 was used to graphically present the results.

### Model evaluation

**Fig. 2:**
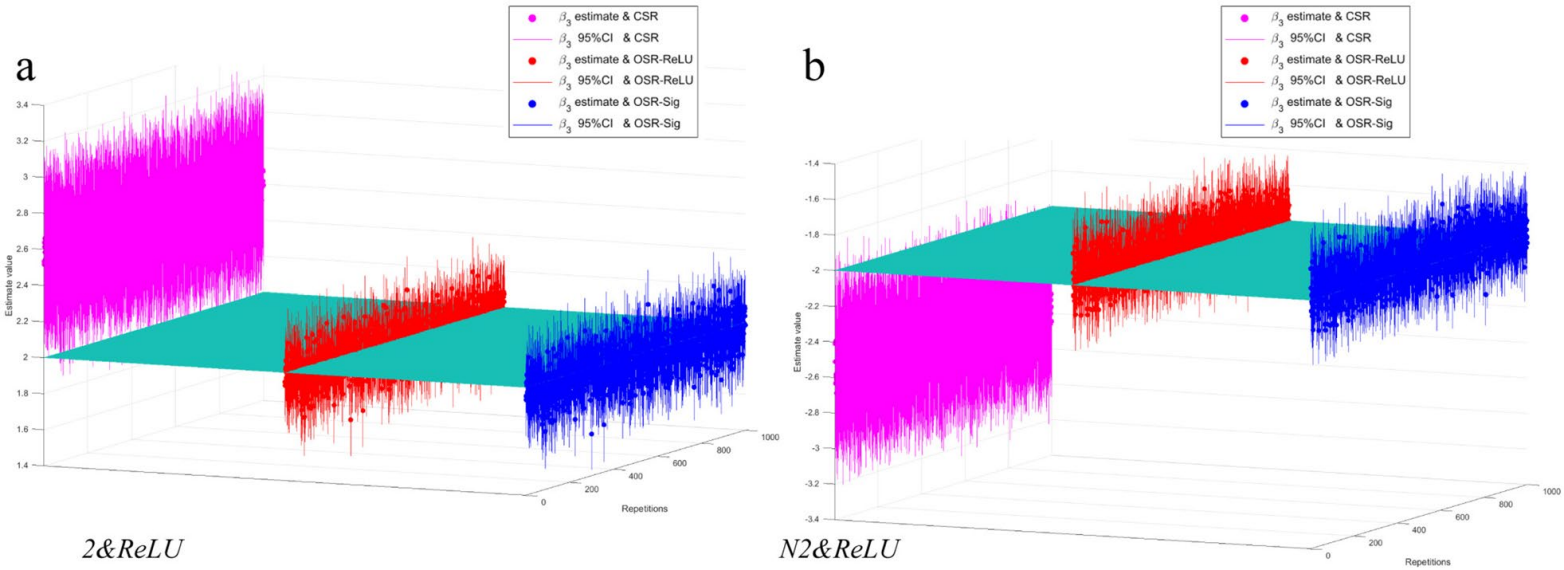
Assignment of F(t)× "intervention" and F(t)× "post-time" during the lag period for different lag lengths L. ${\widehat\beta}_3$ and corresponding 95% CIs with different models with 1000 simulation repetitions. Panel **a** and **b** plot the ${\widehat{\beta }}_{3}$ and corresponding 95% CIs with different models. The outcome time series of Panel **a** and **b** are *2&ReLU* and *N2&ReLU* type with lag length *L* = 4 and adding white noises $N\left(0,{3}^{2}\right)$. Different colors represent different models: pink represents the CSR model, red represents the OSR-ReLU model, and blue represents the OSR-Sig model. The horizontal plane represents the true value of the long-term effect of the intervention on the outcome variable for that type (+ 2 or -2). The dots in the figure represent the point estimates ${\widehat{\beta }}_{3}$ of the long-term effects, and the vertical lines represent the 95% CIs for the corresponding estimates

In this study, the performance of models was judged based on a range of criteria in estimating the long-term impacts of intervention (${\widehat{\beta }}_{3}$ in the models). Figure 2 shows the results of different models for estimating the long-term impact ${\widehat{\beta }}_{3}$ of the intervention. The performance of the models was evaluated based on several metrics, including the bias of the estimates, mean relative error, mean square error, mean width of the 95% CI and coverage rate of the 95% CI. These metrics are defined as follows:

- **Bias of estimates**

$$E\left[{\widehat{\beta }}_{3}\right]-{\beta }_{3}=\frac{1}{n}\sum\limits_{i=1}^{n}{\widehat{\beta }}_{3,i}-{\beta }_{3}$$

- **Mean relative error (MRE)**

$$E\left[\left|\frac{{\widehat{\beta }}_{3}-{\beta }_{3}}{{\beta }_{3}}\right|\times 100\%\right]=\frac{1}{n}\sum\limits _{i=1}^{n}\left|\frac{{\widehat{\beta }}_{3,i}-{\beta }_{3}}{{\beta }_{3}}\right|\times 100\%$$

- **Mean square error (MSE)**

$$E\left[{\left({\widehat{\beta }}_{3}-{\beta }_{3}\right)}^{2}\right]=\frac{1}{n}\sum\limits_{i=1}^{n}{\left({\widehat{\beta }}_{3,i}-{\beta }_{3}\right)}^{2}$$

- **Mean width of 95% CI**

$$E\left[{{\widehat{\beta }}_{3_{uup}}}-{{\widehat{\beta }}_{3_{low}}}\right]=\frac{1}{n}\sum\limits_{i=1}^{n}{{\widehat{\beta }}_{3,i_{uup}}}-{{\widehat{\beta }}_{3,i_{low}}}$$

- **Coverage rate of 95% CI**

$$Pr\left({{\widehat{\beta }}_{3_{low}}}\le {\beta }_{3}\le {{\widehat{\beta }}_{3_{uup}}}\right)=\frac{1}{n}\sum\limits_{i=1}^{n}\left\{\begin{array}{l}1, if {{\widehat{\beta }}_{3,i_{low}}}\le {\beta }_{3}\le {{\widehat{\beta }}_{3,i_{uup}}};\\ 0, else. \end{array}\right.$$

We can examine the bias of parameter estimation for different models by observing the distribution figure of parameter estimation ${\widehat{\beta }}_{3}$ under all simulation scenarios. Simultaneously, we learn how the parameter estimation bias changes as the settings changed in different simulation scenarios. $n$ is the number of simulation repetitions (in this study, $n=\text{1,000}$).

${\beta }_{3}$ is the true value of the long-term impact of the intervention, which is set in advance during the data simulation (+ 2 or -2). ${\widehat{\beta }}_{3,i}$ is the point estimate of the model for long-term impacts at the $i$-th simulation repetition. ${\widehat{\beta}_{3,i_{uup}}}$ and ${{\widehat\beta}_{3,i_{low}}}$ are the upper and lower limits of the 95% confidence interval at the $i$-th simulation repetition, respectively.

## Results

### Bias of estimates

**Fig. 3:**
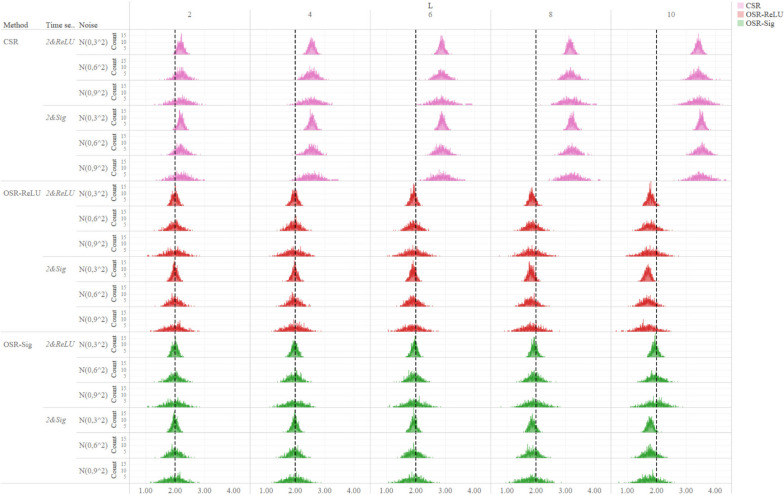
Distributions of long-term impact estimates (${\widehat{{\beta }}}_{3}$) calculated by three methods. The figure plots distributions of long-term impact estimates ${\widehat{\beta }}_{3}$ when the impact true value of long-term impact is 2. Different colors represent different models: pink represents the CSR model, red represents the OSR-ReLU model, and green represents the OSR-Sig model. The vertical black dotted line represents the true value (+ 2). The horizontal axis at the bottom of the figure indicates the axis of the parameter estimate ${\widehat{\beta }}_{3}$, and the vertical axis shows the number of parameters estimates ${\widehat{\beta }}_{3}$ in a specific interval during 1,000 simulation repetitions

Figure 3 presented the distributions of long-term impact estimates specific to scenarios where the true value of the long-term impact was + 2 (Supplementary Material Fig S2 was specific to the scenarios where the true value of long-term impact was − 2). Both OSR-ReLU and OSR-Sig yielded, especially the OSR-Sig, approximately unbiased estimates of long-term impact across nearly all simulation scenarios. In contrast, CSR provided biased estimates of the long-term impact, and the deviation between the estimates and the true value increased with the increase of the lag length ($L$). For all three models, as the degree of fluctuation in the outcome time series increased, the distribution of parameter estimation tended to be flat.

### Mean relative error (MRE)

**Fig. 4:**
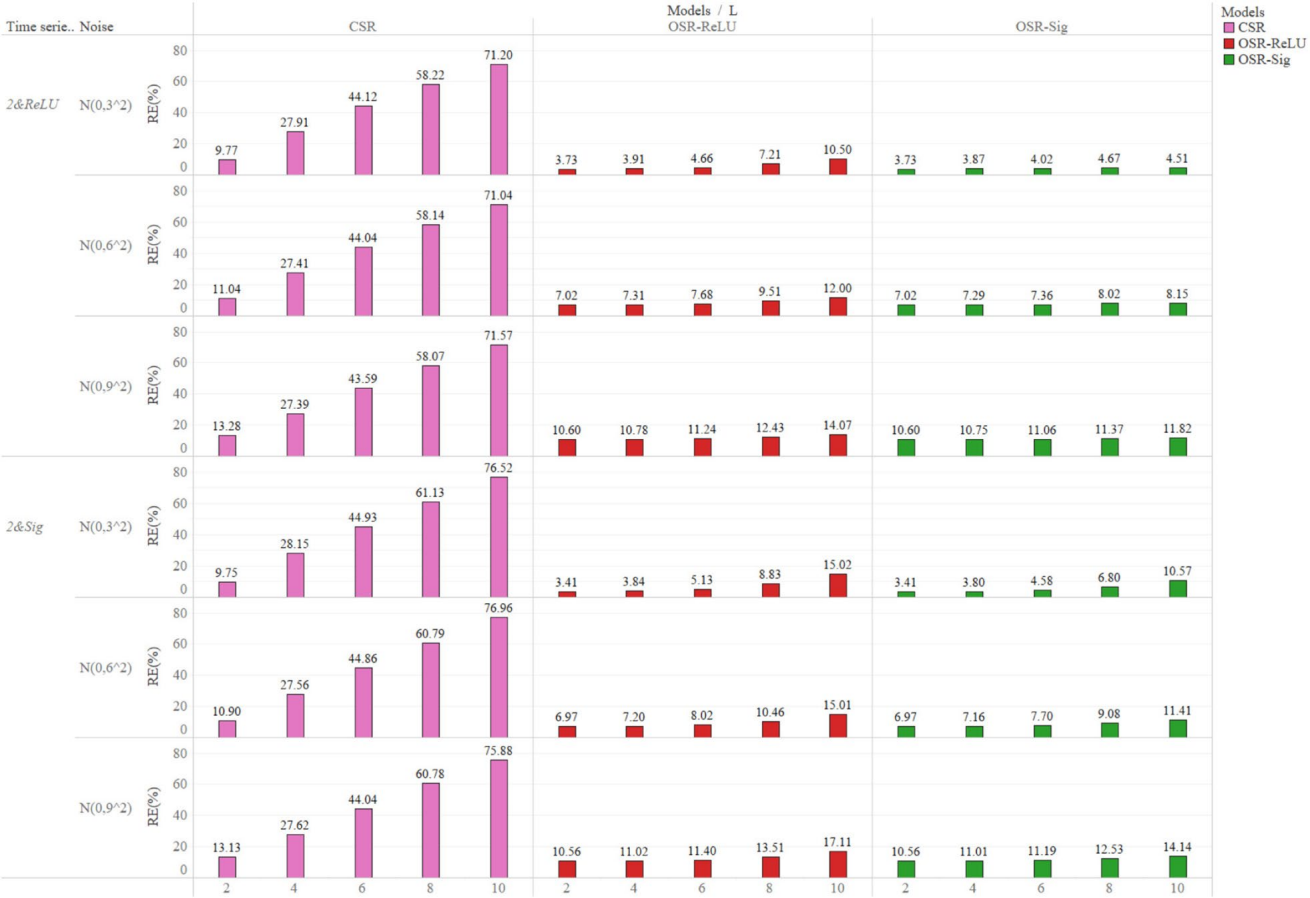
Mean relative error (%) for positive impact simulation scenarios. The horizontal axis at the bottom of the figure represents the lag length ($L$). Mean relative error (%) for negative impact simulation scenarios is shown in Fig S3 of the Supplementary Material

On average, the MRE for OSR-ReLU (9.27%) and OSR-Sig (8.08%) was significantly lower than that of CSR (43.29%) from Table S1. As demonstrated in Table S1, Fig. 4 and Fig S3, with increasing lag length, the average MRE for CSR experienced a substantial rise, jumping from 11.38% ($L$=2) to 73.58% ($L$=10), an increase of nearly 6.5 times. In contrast, the average MRE for both OSR-ReLU and the OSR-Sig remained stable or rose slightly, from 7.13% ($L$=2) to 13.76% ($L$=10) for the OSR-ReLU and from 7.13% ($L$=2) to 9.79% ($L$=10) for the OSR-Sig.

Although the MRE for both OSR-ReLU and the OSR-Sig increased slightly with the rise fluctuation degrees of the outcome time series, from 6.59% ($\sigma$=3) to 12.19% ($\sigma$=9) for the OSR-ReLU and from 4.90% ($\sigma$=3) to 11.42% ($\sigma$=9) for the OSR-Sig, their MRE values were considerably lower than that of CSR. The MRE for CSR remained around 43% as $\sigma$ changed from three to nine.

### Mean square error (MSE)

**Fig. 5:**
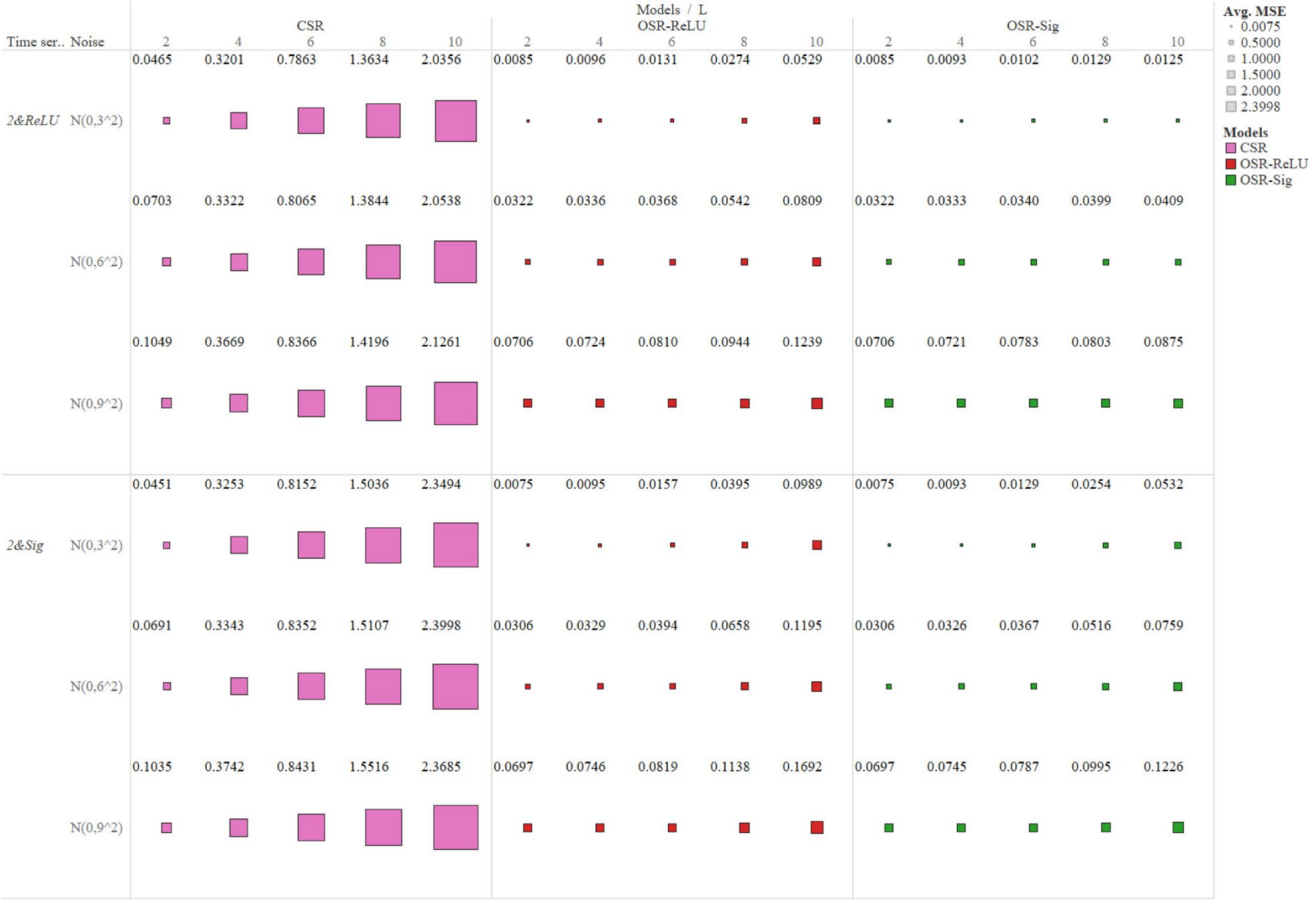
Mean square error for positive impact simulation scenarios. The horizontal axis at the bottom of the figure represents the lag length ($L$). Mean square error for negative impact simulation scenarios is shown in Fig S4 of the Supplementary Material

As shown in Table S2, the average MSE for OSR-ReLU (0.0579) and OSR-Sig (0.0459), was considerably lower than that of CSR (0.9792). As shown in Table S2, Fig. 5 and Fig S4, the MSE for CSR increased significantly with the rise in lag length, moving from 0.0745 ($L$=2) to 2.2056 ($L$=10). In contrast, the MSE for OSR-ReLU rose marginally (from 0.0373 with $L$=2 to 0.1048 with $L$=10), and similarly for OSR-Sig, it increased slightly (from 0.0373 with $L$=2 to 0.0622 with $L$=10).

### Mean width of 95% CI

**Fig. 6:**
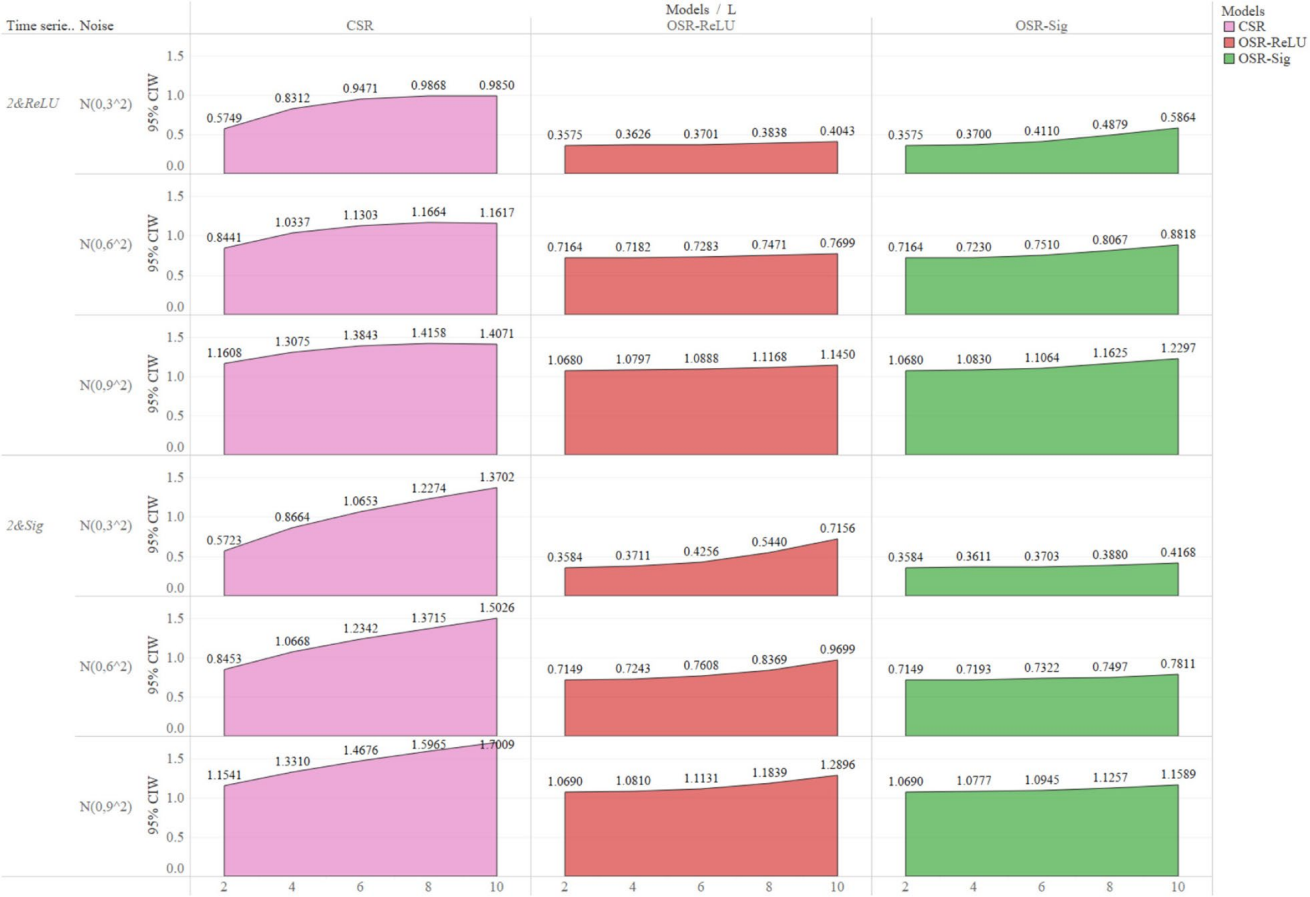
Mean width of 95% CI for positive impact simulation scenarios. The horizontal axis at the bottom of the figure represents the lag length ($L$). Mean width of 95% CI for negative impact simulation scenarios is shown in Fig S5 of the Supplementary Material

The mean widths of the 95% CI for OSR-ReLU (0.7668) and OSR-Sig (0.7607) were lower than that for CSR (1.1481) (Table S3, Fig. 6 and Fig S5). As the lag length increased, the mean width of the 95% CI for both OSR-ReLU and OSR-Sig remained nearly unchanged, especially when the lag length was less than six. In contrast, the mean width of the 95% CI for CSR increased slightly from 1.1580 ($L$=2) to 1.5300 ($L$=10). With an increase in the degree of fluctuation of the outcome time series, the mean width of the 95% CI for the three models increased slightly.

### Coverage rate of 95% CI

**Fig. 7:**
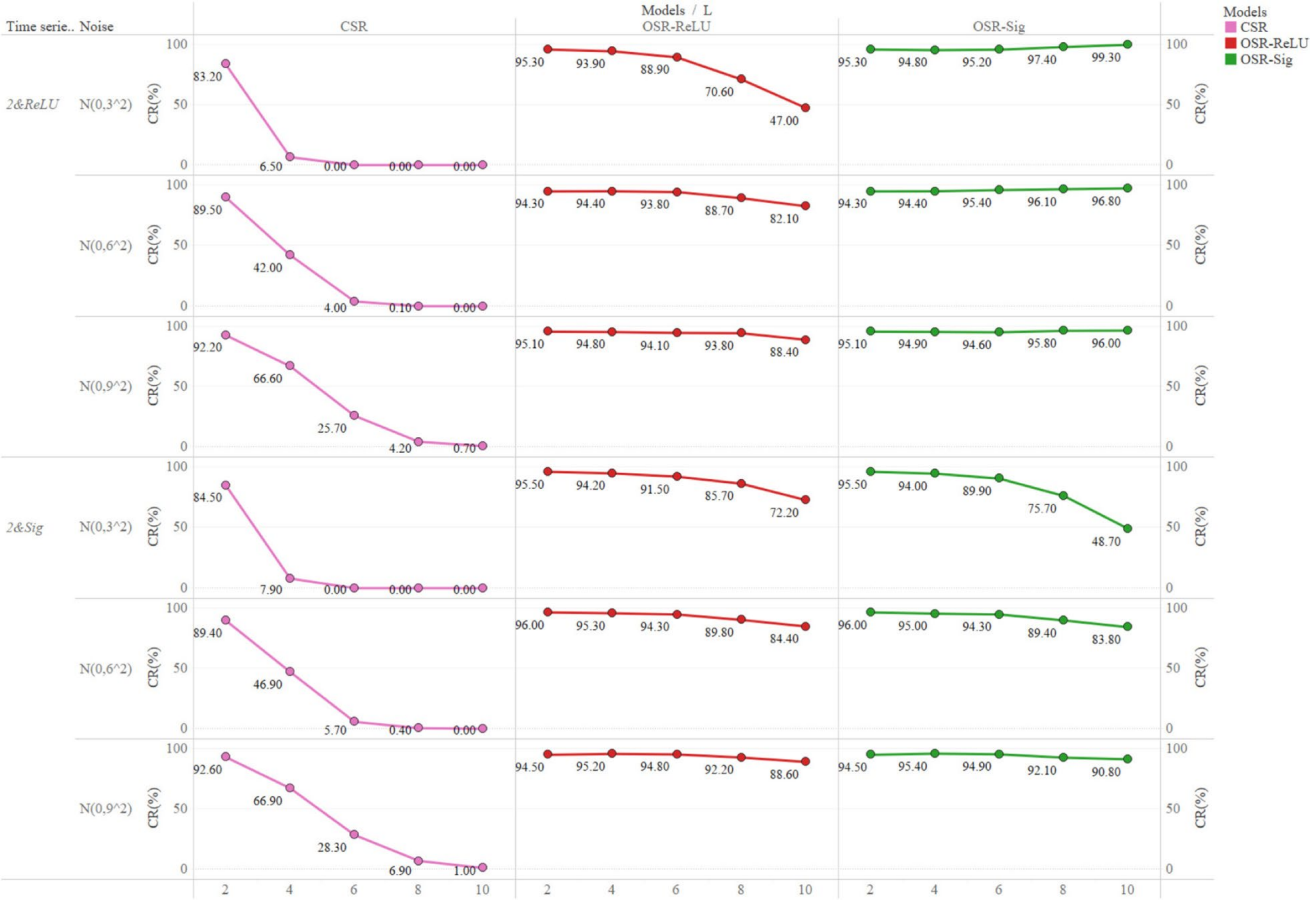
Coverage rate (%) of 95% CI for positive impact simulation scenarios. The horizontal axis at the bottom of the figure represents the lag length ($L$). Coverage rate (%) of 95% CI for negative impact simulation scenarios is shown in Fig S6 of the Supplementary Material

Figure 7 and Fig S6 displayed the coverage rates of the 95% CIs for the true values (+ 2 & -2) across various simulation scenarios and further delineated how the coverage rates changed with increasing lag length $L$ and and fluctuation degree $\sigma$ of the outcome time series, capturing distinct trends in coverage rates among different models. For most simulation scenarios, the coverage rate of 95% CI for the three models was less than the nominal 95% level (Fig. 7 and Fig S6). The coverage rate of the 95% CI for CSR was far below the 95% level in all simulation scenarios, and when the lag length reached four or more, their 95% CI had difficulty in covering the corresponding parameter. When the lag period reached ten, the coverage rate of 95% CI for CSR was only 0.23% (Table S4). For OSR-ReLU and OSR-Sig, the overall coverage rate of 95% CI was slightly lower than the nominal coverage rate (95%), but it reached more than 90% for most scenarios. As the lag length increased, the coverage rate of the 95% CI for OSR-ReLU and OSR-Sig declined slowly, especially when the lag length was less than six. Interestingly, the coverage rate of 95% CI for all three models increased when the fluctuation degree of outcome time series increased ($\sigma$ increased from three to nine).

### Model application

To show the application steps of the OSR models, we provide an application example in Supplementary Material and published the corresponding MATLAB code (https://github.com/AlexZhang662/CSRmodel-application-example/tree/main). In the Supplementary Material section: Application Example, we have a detailed description of the data structure and modeling results of this application example.

## Discussion

In this study, to overcome the defect that the CSR cannot model the lag effects of interventions, we proposed OSR models by introducing different activation functions to the CSR. Using the activation function ReLU and Sigmoid function, we established the OSR-ReLU and OSR-Sig models, and we evaluated the accuracy and precision of the estimation of the long-term impact (slope change) of the intervention for OSR-ReLU, OSR-Sig and CSR models based on a range of simulated scenarios. The OSR models yielded approximately unbiased estimates of long-term impact and outperformed the CSR model in terms of accuracy and precision.

The introduction of an activation function that incorporates lags into the model, as expected, can significantly improve the accuracy of the long-term impact estimates of interventions. Both the MRE and MSE of OSR-ReLU and OSR-Sig were significantly lower than those of CSR, and as the lag length increased, the accuracy of the optimized model for long-term impact estimation was also quite robust, especially when *L* was less than six-time points. Compared to CSR, OSR-ReLU and OSR-Sig corrected for the long-term impact parameter estimates of the intervention and obtained an approximately unbiased estimate for almost all scenarios. Direct modeling with the CSR model within the post-intervention phases violated basic linearity requirement for the regression model [28, 29]. As the lag period increased, the linearity requirement becomes more difficult to satisfy, resulting in more biased parameter estimates, as proven by our simulation results. The OSR model adjusts the independent variables of the regression model by introducing activation functions during the lag period that fit the actual characteristics of the data and significantly reduces the bias of the parameter estimates. However, when the intervention has no lag effect, blind use of the OSR model instead leads to worse results. In the Supplementary Material - Baseline simulation, we additionally designed baseline simulation scenarios with no lags but satisfying all basic CSR assumptions to demonstrate the potential loss in efficiency of OSR models. Therefore, we should be more cautious about specifying different models.

OSR-ReLU and OSR-Sig also outperformed CSR in terms of precision, with the corresponding 95% CI having narrower mean widths than CSR while having higher coverage rates. In fact, a model with a wider 95% CI was more likely to cover the parameters [30, 31]. However, the CSR model’s biased estimates of parameters had a detrimental impact on ${\widehat{\beta }}_{3}$ with higher MRE and MSE, which in turn resulted in a lower coverage rate of 95% CI than that of the OSR-ReLU and the OSR-Sig, and far less than the nominal 95% level [25]. Ideally, the coverage rate of 95% CI should be at the nominal 95% level with no bias; [32, 33] however, the coverage rate of 95% CI for OSR-ReLU (88.82%) and OSR-Sig (92.31%) was slightly below this level, which indicating that optimized models provided approximately unbiased estimates. This is because OSR models alleviate the heteroscedasticity of the error term without completely eliminating it. Thus, an estimation model for the full unbiasedness of the long-term impact may require further research.

Our results showed that as the fluctuation degree ($\sigma$) of the outcome time series increased, the mean width of 95% CI and the MSE of the estimates increased, but the corresponding coverage rate of the 95% CI decreased. This suggests that a wider 95% CI is more effective in covering the parameters, despite the increased deviation of the estimates from the true values in our simulation scenarios. Occasionally, the coverage rate of the parameters is crucial for comprehending the parameter distributions [34, 35]. We argued that the correct method should minimize the error while ensuring high estimation precision, similar to the OSR-ReLU and OSR-Sig proposed in this study.

### Strengths

Employing different activation functions (linear ReLU and nonlinear Sigmoid function) to model different intervention lag patterns is a novel strength of this study. The corresponding optimized models can effectively describe the lag process and surpass the CSR model in both accuracy and precision when estimating of the long-term impact of interventions, especially when intervention lag effects are present. Our simulation scenarios were inspired by real-world ITS studies, [36, 37] including the intervention’s positive or negative impacts, different lag patterns, different lag lengths, different fluctuation degrees of outcome time series, and 60 different scenarios altogether, which provided a comprehensive description of the interrupted time series analysis in many cases.

### Limitations

There are several limitations of this study. First, for each simulation scenario, we aim to maintain the Monte Carlo Standard Error (MCSE) below 0.5% for all potential coverage rate values [38, 39]. Although more repetitions would result in a smaller MCSE, it remains to be determined whether the current 1,000 repetitions are sufficient to maintain the MCSE below 0.5%, or how many repetitions are sufficient. Second, all outcome time-series’ changes resulted from a random number generator, which limited the applicability of our findings, as with all simulation studies. Third, considering the potential loss in efficiency of OSR models when the intervention has no lag effect, it is critical to specify between CSR and OSR, which is also a difficulty highlighted in Discussion section of this study. Four, the parameter (lag length: $L$) used in the OSR models was known and consistent with the lag length $L$ of the simulated outcome time series. However, in practical studies, selecting the activation function and the parameter $L$ is major challenge in the statistical analysis of OSR models. Two possible approaches are the implementation-driven and the data-driven approaches. In the application example in Supplementary Material - Application Example, we conducted a preliminary exploration of these two approaches. In addition, the robustness of the difference between parameter $L$ incorporated in the OSR model and the actual lag length ${L}_{a}$ of the intervention warrants further investigation.

## Conclusion

To address the limitation of CSR, which fails to model the lag effects of interventions, we propose two optimized models, OSR-ReLU and OSR-Sig. These models employ a linear ReLU function and a nonlinear Sigmoid function, respectively, to represent different lag patterns. Based on simulated data, both OSR-ReLU and OSR-Sig outperformed the CSR model in terms of both accuracy and precision, and yielded approximately unbiased estimates. Our optimized models provide a more comprehensive and effective tool for evaluating intervention effects under an interrupted time series design.

### Supplementary Information

**Additional file 1.**

## Data Availability

The datasets used and/or analyzed during the current study are available from the corresponding author on reasonable request.
