# Supplementary material for: Introducing activation functions into segmented regression model to address lag effects of interventions
Source: BMC Med Res Methodol. 2023 Nov 24;23:277. doi: 10.1186/s12874-023-02098-x (PMC10668529; doi:10.1186/s12874-023-02098-x)
Supplement: Supplementary file 1 — Additional file 1. [file 12874_2023_2098_MOESM1_ESM.docx]

# Supplementary Material

- **Explanation 1.**

We denote $f^{\left( 1 \right)}$ and $f^{\left( 2 \right)}$ as the value of activation functions ReLU and Sigmoid when the lag length is 2, and it can be proved that the OSR-ReLU and OSR-Sig models are equivalent.

The following are expressions for the activation function ReLU and Sigmoid:

1. Linear ReLU: $f_{ReLU}\left( t \right)=\frac{1}{\text{L}}*t; T_{0}\leq t\leq T_{0}+L$;
2. Nonlinear Sigmoid: $f_{Sig}\left( t \right)=\frac{a}{\text{1+}e^{-t}}+b$, subject to $\left\{ \begin{aligned} \frac{\text{a}}{\text{1+}e^{-\left( -\frac{\text{L}}{2} \right)}}+\text{b}=0, \\ \frac{\text{a}}{\text{1+}e^{-\frac{\text{L}}{2}}}+\text{b}=1. \end{aligned} \right.$

For the OSR-ReLU model, $f_{ReLU}^{(1)}=f_{ReLU}\left( \frac{L}{\text{2}} \right)=\frac{1}{\text{L}}*\frac{L}{\text{2}}=\frac{1}{\text{2}}$; $f_{ReLU}^{(2)}=f_{ReLU}\left( L \right)=\frac{1}{\text{L}}*L=1$.

For the OSR-Sig model, $f_{Sig}^{(1)}=f_{Sig}\left( 0 \right)=\frac{a}{\text{1+}e^{-0}}+b=\frac{a}{\text{2}}+b$.

Considering $\left\{ \begin{aligned} \frac{\text{a}}{\text{1+}e^{-\left( -\frac{\text{L}}{2} \right)}}+\text{b}=0, \\ \frac{\text{a}}{\text{1+}e^{-\frac{\text{L}}{2}}}+\text{b}=1. \end{aligned} \right.$ $\Longrightarrow\left\{ \begin{aligned} \frac{\text{a}}{\text{1+}e^{\frac{\text{L}}{2}}}+\text{b}=0,① \\ \frac{a*e^{\frac{\text{L}}{2}}}{e^{\frac{\text{L}}{2}}\text{+}1}+\text{b}=1. ② \end{aligned} \right.$, then $\frac{\mathbf{①+②}}{\boldsymbol{2}}$**:** $\frac{1}{2}*\left[ \frac{a*\left( e^{\frac{\text{L}}{2}}+1 \right)}{e^{\frac{\text{L}}{2}}\text{+}1}+2*b \right] =\frac{1}{2}$, then we have $f_{Sig}^{(1)}=\frac{a}{\text{2}}+b=\frac{1}{2}=f_{ReLU}^{(1)}$. Besides $f_{Sig}^{(2)}=f_{Sig}\left( \frac{L}{\text{2}} \right)=\frac{\text{a}}{\text{1+}e^{-\frac{\text{L}}{2}}}+\text{b}=1=f_{ReLU}^{(2)}$.

Then we have $\left\{ \begin{aligned} f_{ReLU}^{(1)}=f_{Sig}^{(1)}=\frac{1}{2}; \\ f_{ReLU}^{(2)}=f_{Sig}^{(2)}=1. \end{aligned} \right.$


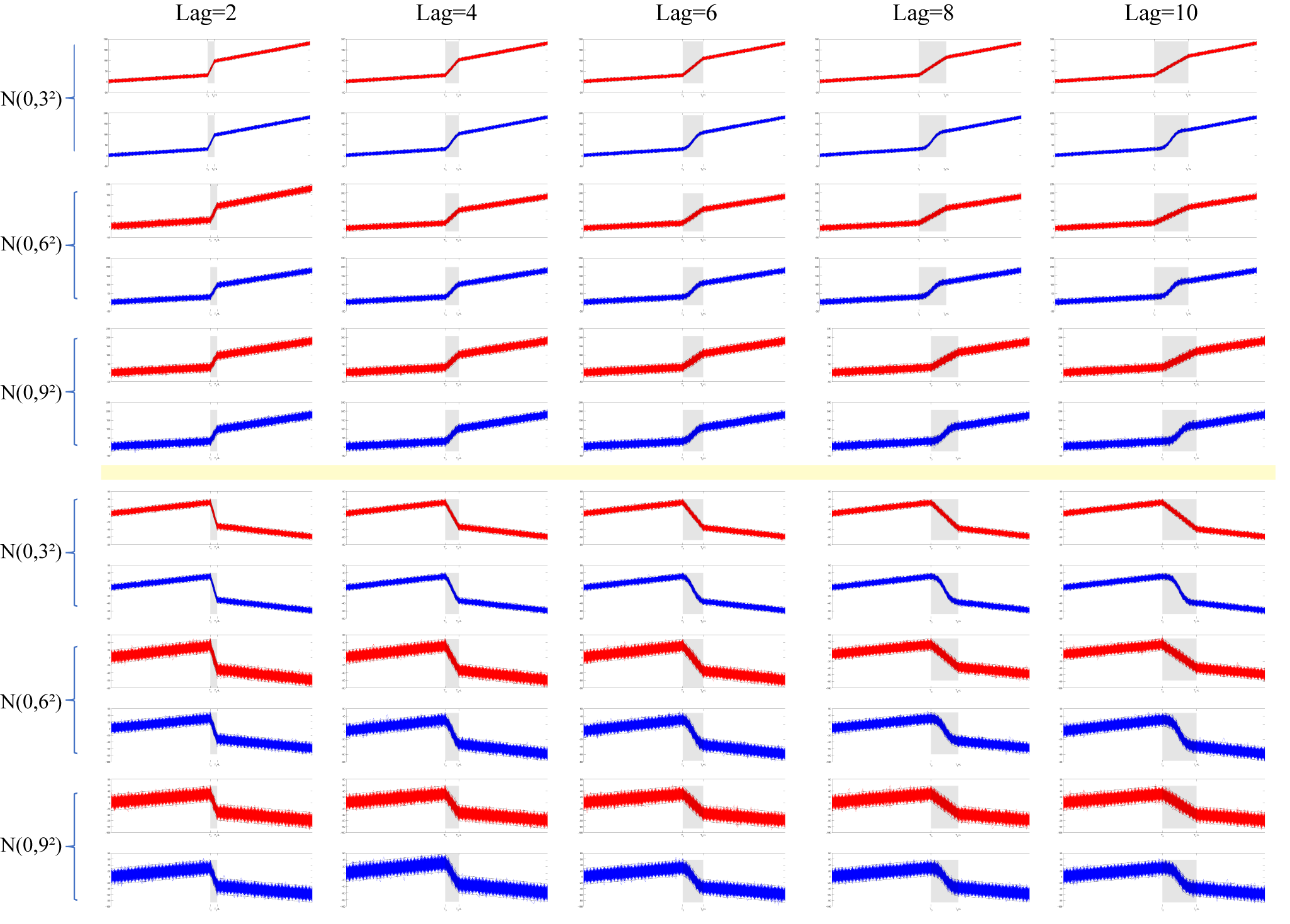


**Fig S1. Outcome time series simulation scenarios.**

The different colors represent different patterns of intervention lags, red for the linear lag pattern (ReLU) and blue for the nonlinear lag pattern (Sigmoid). The top half of the figure represents the scenarios where the simulating intervention effect is positive (+2) for the time series and the bottom half of the figure represents the scenarios where the simulating intervention is negative (-2) for the outcome time series. Different lag lengths and different levels of noise fluctuations correspond to the scenarios shown in the legend. For one specific characteristic scenario, we repeat the simulation 1,000 times, and the specific panel shows 1,000 outcome time series.


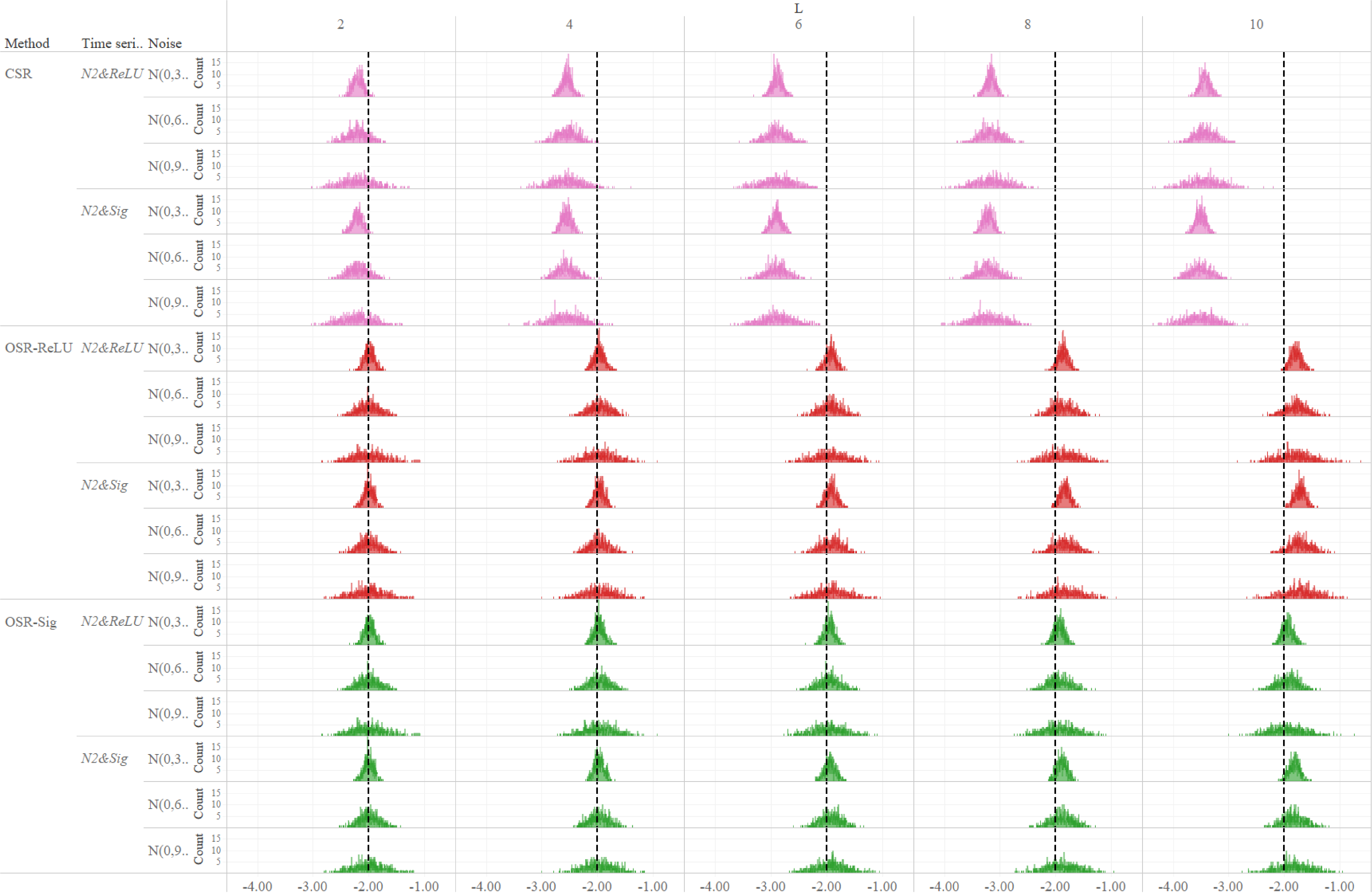


**Fig S2. Distributions of long-term impact estimates** ${\hat{\boldsymbol{\beta}}}_{\boldsymbol{3}}$ **calculated from three methods.**

The figure plots distributions of long-term impact estimates $\hat{\beta}_{3}$ when the impact true value of long-term impact is -2. Different colors represent different models, pink for the CSR model, red for the OSR-ReLU model, and green for the OSR-Sig model. The vertical black dotted line represents the true value (-2). The horizontal axis at the bottom of the figure indicates the axis of the parameter estimate $\hat{\beta}_{3}$, and the vertical axis shows the number of parameters estimates $\hat{\beta}_{3}$ in a specific interval during 1,000 simulation repetitions.

**Table S1. Mean relative error (%) for different lag lengths and fluctuation degree of outcome time series.**

| **Model** | **White noise** | **Lag length** | | | | | |
| --- | --- | --- | --- | --- | --- | --- | --- |
|  |  | 2 | 4 | 6 | 8 | 10 | Mean |
| **CSR** | $N\left( 0,3^{2} \right)$ | 9.74 | 27.88 | 44.48 | 59.54 | 73.50 | 43.03 |
|  | $N\left( 0,6^{2} \right)$ | 10.98 | 27.57 | 44.42 | 59.50 | 73.64 | 43.22 |
|  | $N\left( 0,9^{2} \right)$ | 13.42 | 27.68 | 43.98 | 59.34 | 73.61 | 43.61 |
|  | Mean | 11.38 | 27.71 | 44.29 | 59.46 | 73.58 | 43.29 |
| **OSR-ReLU** | $N\left( 0,3^{2} \right)$ | 3.61 | 3.87 | 4.96 | 7.90 | 12.59 | 6.59 |
|  | $N\left( 0,6^{2} \right)$ | 7.06 | 7.11 | 7.90 | 9.84 | 13.32 | 9.05 |
|  | $N\left( 0,9^{2} \right)$ | 10.71 | 10.75 | 11.31 | 12.81 | 15.37 | 12.19 |
|  | Mean | 7.13 | 7.24 | 8.06 | 10.18 | 13.76 | 9.27 |
| **OSR-Sig** | $N\left( 0,3^{2} \right)$ | 3.61 | 3.82 | 4.37 | 5.54 | 7.18 | 4.90 |
|  | $N\left( 0,6^{2} \right)$ | 7.06 | 7.09 | 7.58 | 8.46 | 9.46 | 7.93 |
|  | $N\left( 0,9^{2} \right)$ | 10.71 | 10.73 | 11.10 | 11.83 | 12.72 | 11.42 |
|  | Mean | 7.13 | 7.21 | 7.68 | 8.61 | 9.79 | 8.08 |


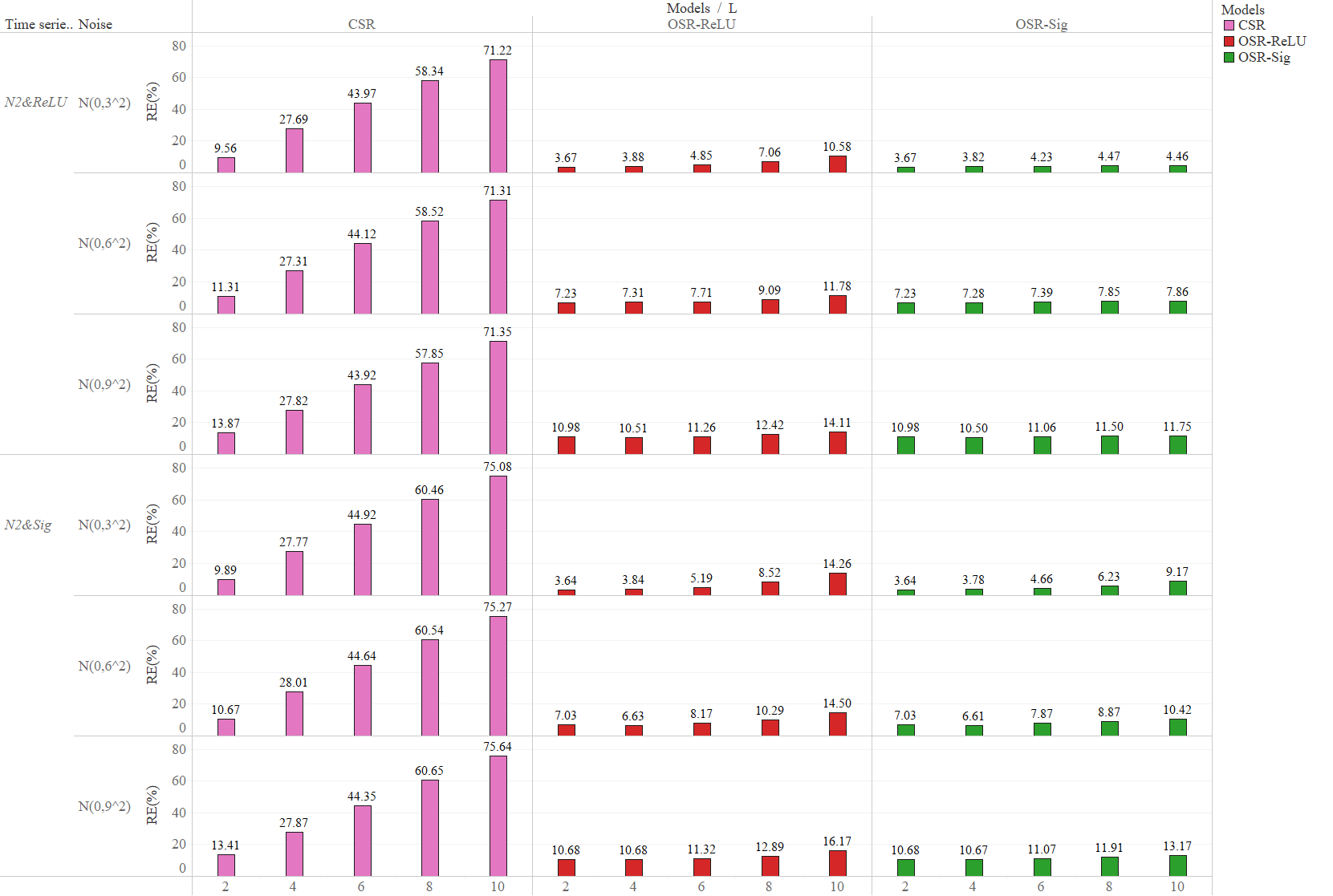


**Fig S3. Mean relative error (%) for negative impact simulation scenarios.**

The horizontal axis at the bottom of the figure represents the lag length ($L$).

**Table S2. Mean square error for different lag lengths and fluctuation degree of outcome time series.**

| **Model** | **White noise** | **Lag length** | | | | | |
| --- | --- | --- | --- | --- | --- | --- | --- |
|  |  | 2 | 4 | 6 | 8 | 10 | Mean |
| **CSR** | $N\left( 0,3^{2} \right)$ | 0.0457 | 0.3192 | 0.7996 | 1.4266 | 2.1711 | 0.9524 |
|  | $N\left( 0,6^{2} \right)$ | 0.0695 | 0.3341 | 0.8201 | 1.4494 | 2.2038 | 0.9754 |
|  | $N\left( 0,9^{2} \right)$ | 0.1083 | 0.3725 | 0.8454 | 1.4815 | 2.2419 | 1.0099 |
|  | Mean | 0.0745 | 0.3419 | 0.8217 | 1.4525 | 2.2056 | 0.9792 |
| **OSR-ReLU** | $N\left( 0,3^{2} \right)$ | 0.0081 | 0.0094 | 0.0147 | 0.0324 | 0.0739 | 0.0277 |
|  | $N\left( 0,6^{2} \right)$ | 0.0316 | 0.0319 | 0.0387 | 0.0588 | 0.0977 | 0.0517 |
|  | $N\left( 0,9^{2} \right)$ | 0.0722 | 0.0722 | 0.0811 | 0.1026 | 0.1427 | 0.0942 |
|  | Mean | 0.0373 | 0.0378 | 0.0448 | 0.0646 | 0.1048 | 0.0579 |
| **OSR-Sig** | $N\left( 0,3^{2} \right)$ | 0.0081 | 0.0092 | 0.0118 | 0.0180 | 0.0301 | 0.0154 |
|  | $N\left( 0,6^{2} \right)$ | 0.0316 | 0.0317 | 0.0359 | 0.0448 | 0.0549 | 0.0398 |
|  | $N\left( 0,9^{2} \right)$ | 0.0722 | 0.0720 | 0.0783 | 0.0883 | 0.1016 | 0.0825 |
|  | Mean | 0.0373 | 0.0376 | 0.0420 | 0.0504 | 0.0622 | 0.0459 |


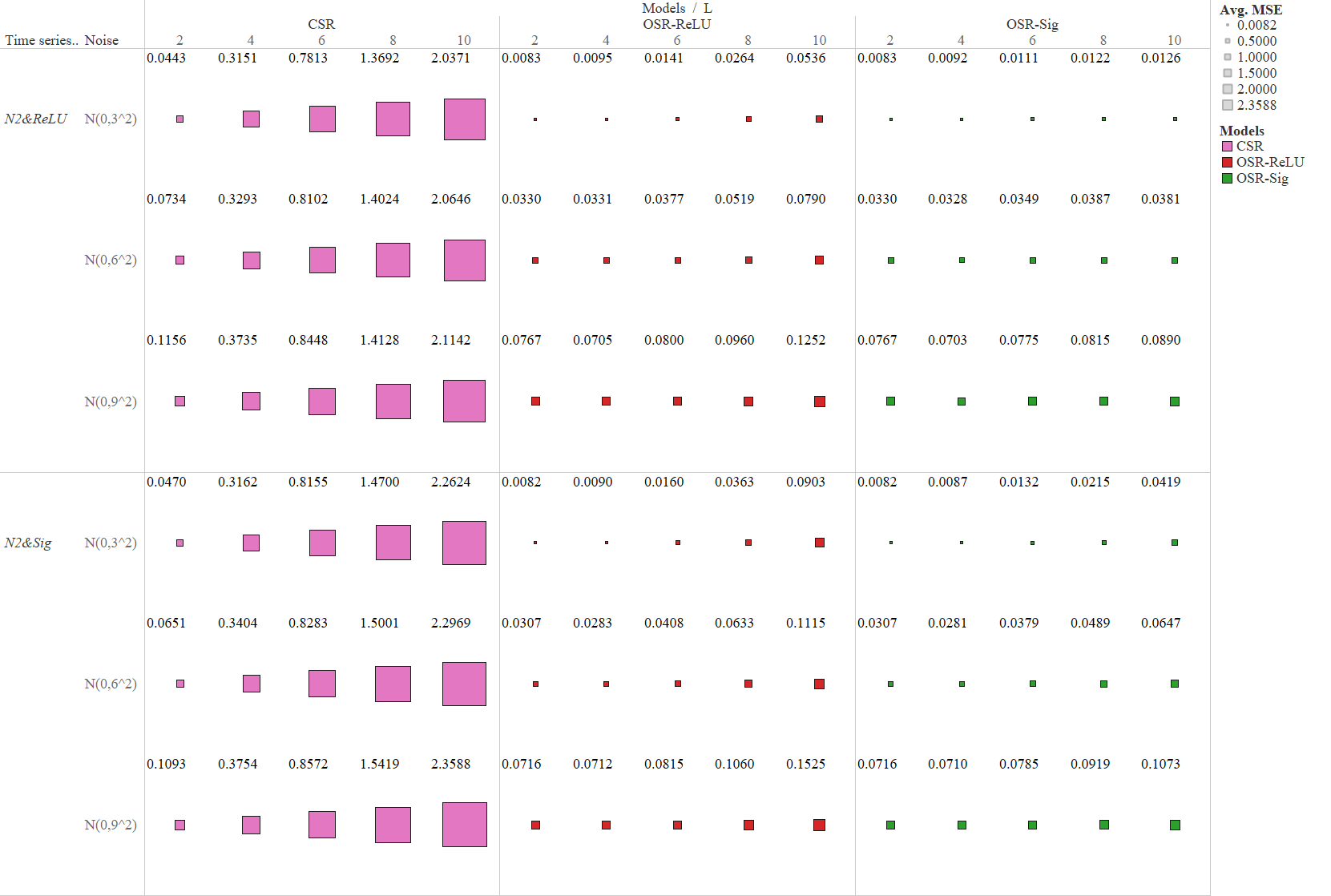


**Fig S4. Mean square error for negative impact simulation scenarios.**

The horizontal axis at the bottom of the figure represents the lag length ($L$).

**Table S3. Mean width of 95% CI for different lag lengths and fluctuation degree of outcome time series.**

| **Model** | **White noise** | **Lag length** | | | | | |
| --- | --- | --- | --- | --- | --- | --- | --- |
|  |  | 2 | 4 | 6 | 8 | 10 | Mean |
| **CSR** | $N\left( 0,3^{2} \right)$ | 0.5716 | 0.8468 | 1.0003 | 1.0942 | 1.1524 | 0.9331 |
|  | $N\left( 0,6^{2} \right)$ | 0.8428 | 1.0498 | 1.1792 | 1.2563 | 1.3086 | 1.1273 |
|  | $N\left( 0,9^{2} \right)$ | 1.1579 | 1.3177 | 1.4207 | 1.4936 | 1.5302 | 1.3840 |
|  | Mean | 1.1580 | 1.3180 | 1.4210 | 1.4940 | 1.5300 | 1.1481 |
| **OSR-ReLU** | $N\left( 0,3^{2} \right)$ | 0.3570 | 0.3653 | 0.3939 | 0.4514 | 0.5336 | 0.4202 |
|  | $N\left( 0,6^{2} \right)$ | 0.7149 | 0.7210 | 0.7431 | 0.7843 | 0.8506 | 0.7628 |
|  | $N\left( 0,9^{2} \right)$ | 1.0685 | 1.0800 | 1.0987 | 1.1418 | 1.1983 | 1.1175 |
|  | Mean | 0.7135 | 0.7221 | 0.7452 | 0.7925 | 0.8608 | 0.7668 |
| **OSR-Sig** | $N\left( 0,3^{2} \right)$ | 0.3570 | 0.3647 | 0.3903 | 0.4362 | 0.4982 | 0.4093 |
|  | $N\left( 0,6^{2} \right)$ | 0.7149 | 0.7211 | 0.7419 | 0.7776 | 0.8312 | 0.7573 |
|  | $N\left( 0,9^{2} \right)$ | 1.0685 | 1.0803 | 1.0999 | 1.1399 | 1.1884 | 1.1154 |
|  | Mean | 0.7135 | 0.7220 | 0.7440 | 0.7846 | 0.8393 | 0.7607 |


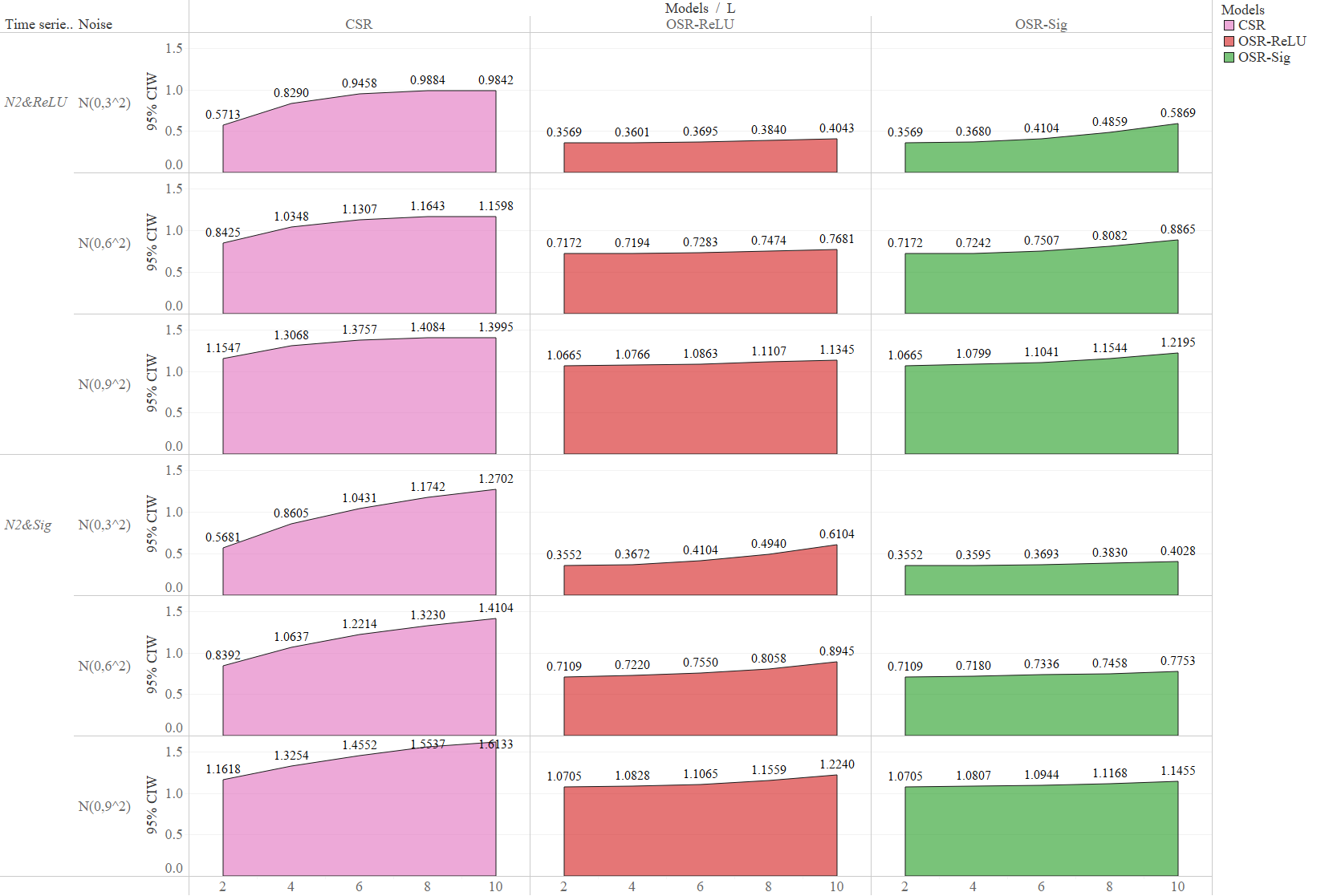


**Fig S5. Mean width of 95% CI for negative impact simulation scenarios.**

The horizontal axis at the bottom of the figure represents the lag length ($L$).

**Table S4. Coverage rate (%) of 95% CI for different lag lengths and fluctuation degree of outcome time series.**

| **Model** | **White noise** | **Lag length** | | | | | |
| --- | --- | --- | --- | --- | --- | --- | --- |
|  |  | 2 | 4 | 6 | 8 | 10 | Mean |
| **CSR** | $N\left( 0,3^{2} \right)$ | 83.73 | 7.25 | 0.00 | 0.00 | 0.00 | 18.20 |
|  | $N\left( 0,6^{2} \right)$ | 89.70 | 43.55 | 4.83 | 0.20 | 0.00 | 27.66 |
|  | $N\left( 0,9^{2} \right)$ | 91.75 | 65.28 | 26.28 | 5.63 | 0.68 | 37.92 |
|  | Mean | 88.39 | 38.69 | 10.37 | 1.94 | 0.23 | 27.92 |
| **OSR-ReLU** | $N\left( 0,3^{2} \right)$ | 94.95 | 94.10 | 89.33 | 77.05 | 56.30 | 82.35 |
|  | $N\left( 0,6^{2} \right)$ | 94.98 | 94.93 | 93.85 | 89.18 | 82.53 | 91.09 |
|  | $N\left( 0,9^{2} \right)$ | 94.60 | 95.00 | 94.10 | 92.65 | 88.78 | 93.03 |
|  | Mean | 94.84 | 94.68 | 92.43 | 86.29 | 75.87 | 88.82 |
| **OSR-Sig** | $N\left( 0,3^{2} \right)$ | 94.95 | 94.53 | 91.98 | 87.50 | 75.80 | 88.95 |
|  | $N\left( 0,6^{2} \right)$ | 94.98 | 94.93 | 94.53 | 92.93 | 91.08 | 93.69 |
|  | $N\left( 0,9^{2} \right)$ | 94.60 | 95.10 | 94.43 | 94.00 | 93.33 | 94.29 |
|  | Mean | 94.84 | 94.85 | 93.64 | 91.48 | 86.73 | 92.31 |


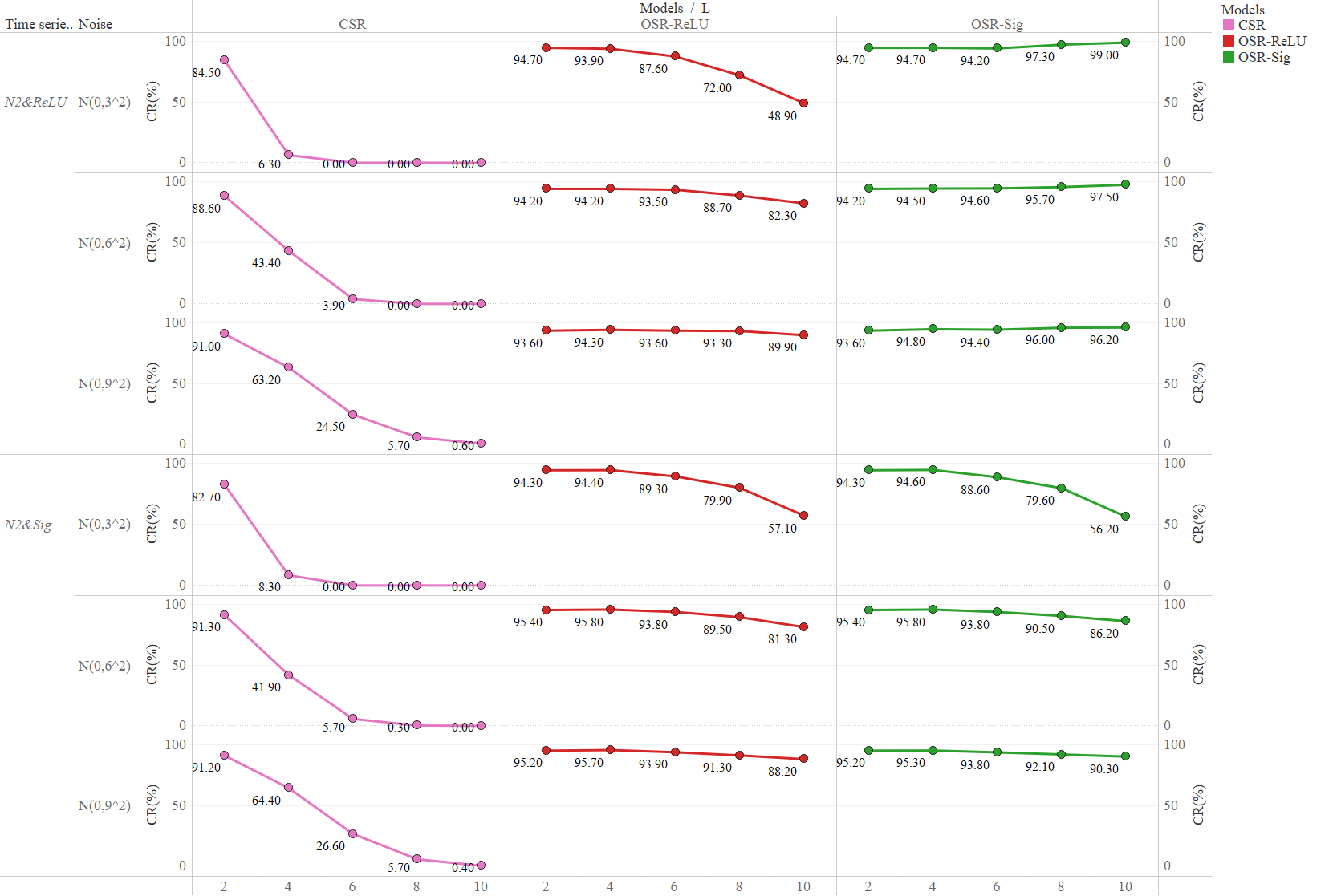


**Fig S6. Coverage rate (%) of 95% CI for negative impact simulation scenarios.**

The horizontal axis at the bottom of the figure represents the lag length ($L$).

- **Application Example:**

We used the raw data from published research [1] entitled ‘[*Effect of Implementing a Free Delivery Service Policy on Women’s Utilization of Facility-Based Delivery in Central Ethiopia: An Interrupted Time Series Analysis*](https://www.hindawi.com/journals/jp/2020/8649598/)’ as an application example of our study. The raw data were included in the Supplementary file of the published study and we can directly download the data from the [*Journal of Pregnancy*](https://www.hindawi.com/journals/jp/2020/8649598/).

Facility delivery services were not commonly used or accessible in Ethiopia. In July 2013, the Ethiopian government put into effect a policy of free delivery services in all public health facilities to encourage mothers to give birth in facilities. The government built a primary healthcare facility in the East Shewa administrative region, which served as the administrative hub for the implementation of the national free delivery service intervention in all public health facilities. Data for 108-time points, comprising facility-based usage of delivery services (72 pre- and 36 post-intervention phases), were available for the nine years between July 2007 and June 2016. The five health facilities' combined monthly birth rate was used as the outcome variable (**Fig S7**).


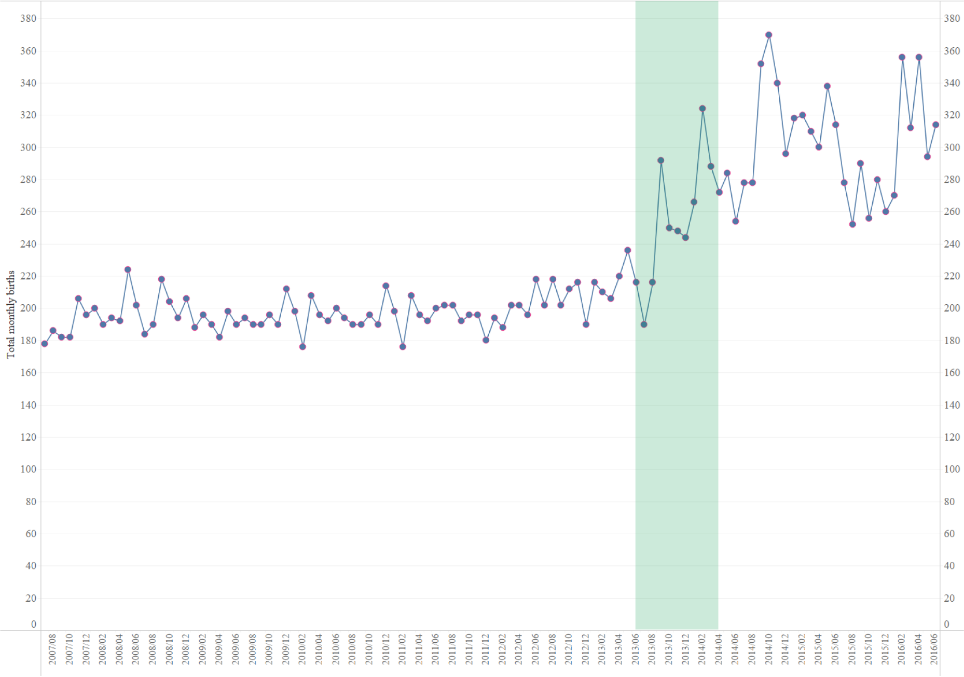


**Fig S7. Time series of total monthly births.**

2007/07 and 2016/06 are the start and end time points of the time series, respectively, and 2013/06 is the nominal intervention time point (free delivery services policy). The shaded part of the figure represents one example scenario of the lag length $L=10$.

The Ethiopian government implemented this nationwide free delivery service intervention. After the formal implementation of the intervention measures in July 2013, the Ethiopian government undertook a series of actions to ensure the comprehensive implementation of this policy intervention. These actions included purchasing emergency vehicles, increasing the number of beds in healthcare facilities, acquiring relevant childbirth equipment, and providing training for healthcare professionals.

Considering the gap between policy advocacy and public awareness, most pregnant women were not immediately aware of the existence of this policy. Furthermore, even among those women in the Sheba region of Ethiopia who were aware of the free delivery service policy and immediately became pregnant upon its formal implementation in July 2013, they gave birth almost 10 months later. Therefore, the intervention effect could not fully manifest at the time of implementation, and considering the lag effect in the generation of intervention impacts was of practical significance. Hence, this intervention was considered an ideal application case for the OSR model.

1. **Step: Selection of the lag length**

There are two possible approaches to the selection of the lag length $L$ and the lag pattern of the intervention effect, namely the implementation-driven approach and the data-driven approach.

Under the implementation-driven approach, researchers can determine the length of the transition period and the lag pattern of the intervention effect (linear: ReLU or nonlinear: Sigmoid) based on the implementation process. In the application example, the lag period can be defined as the time between the start and end of medical staff training, or the gap between policy advocacy and public awareness. For the lag pattern of possible intervention effects, the most appropriate lag pattern based on the training process can also be defined, such as the frequency of training and the number of people per training. In this sense, the parameters set by the implementation-driven approach are consistent with the intervention process and have practical relevance.[2, 3]

Although it is better to use the implementation-driven approach, the relevant information is not always available to the public. Given this, a data-driven approach is an alternative method, which can select the lag length $L$ for OSR models by using the mean squared error (MSE) or other metrics as a measure of goodness of fit.

In our application example, considering the gap between policy advocacy and public awareness, and the length of a woman's pregnancy, we assumed that the maximum range of $L$ was 10 months, i.e., $L_{m}=10$. Among all these possible scenario decision sets ($L=0, 1, 2,\ldots,10$), the selected $L$ were selected by the minimum of MSE the model application.


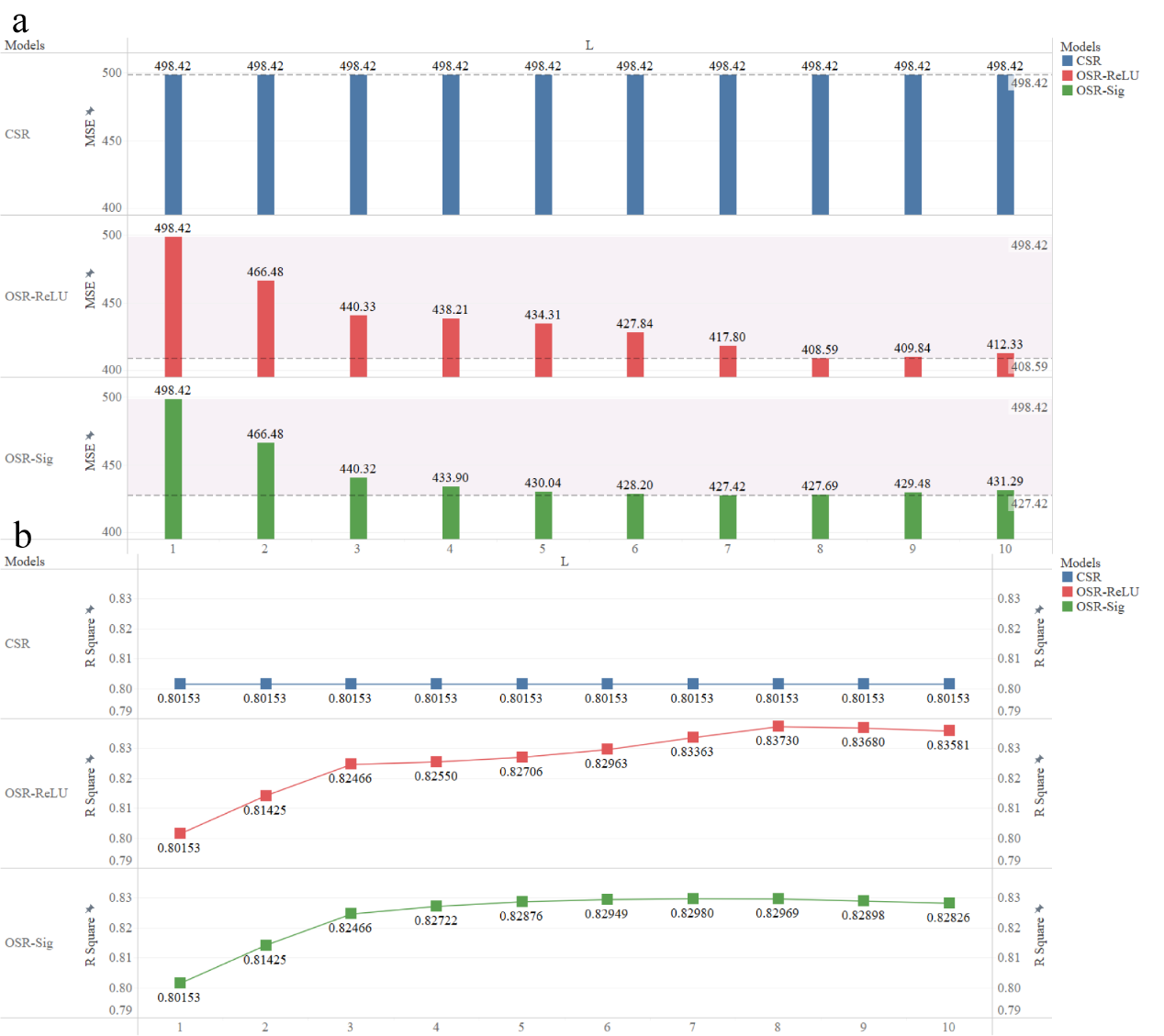


**Fig S8. Mean squared errors and R squared of models with all possible lag lengths.**

From **Fig S8**, we learned that MSEs of OSR models (OSR-ReLU & OSR-Sig) were smaller than those of the CSR model ($L=0$) and the $R^{2}$ (coefficient of determination) of OSR models was larger than those of the CSR model regardless of the lag length $L$, indicating OSR models fitted the data better. Using the minimum MSE as the selection metric, the OSR-ReLU & OSR-Sig models were chosen with different lag lengths $L$. The selected $L$ for OSR-ReLU model was 8 and the selected $L$ for OSR-Sig model was 7, where the MSE of OSR-ReLU reached the minimum MSE value of 408.59.

**Fig S9** shows the fitted CSR and OSR curves of the outcome time series, and their fitting errors, which indicated appropriate fits.


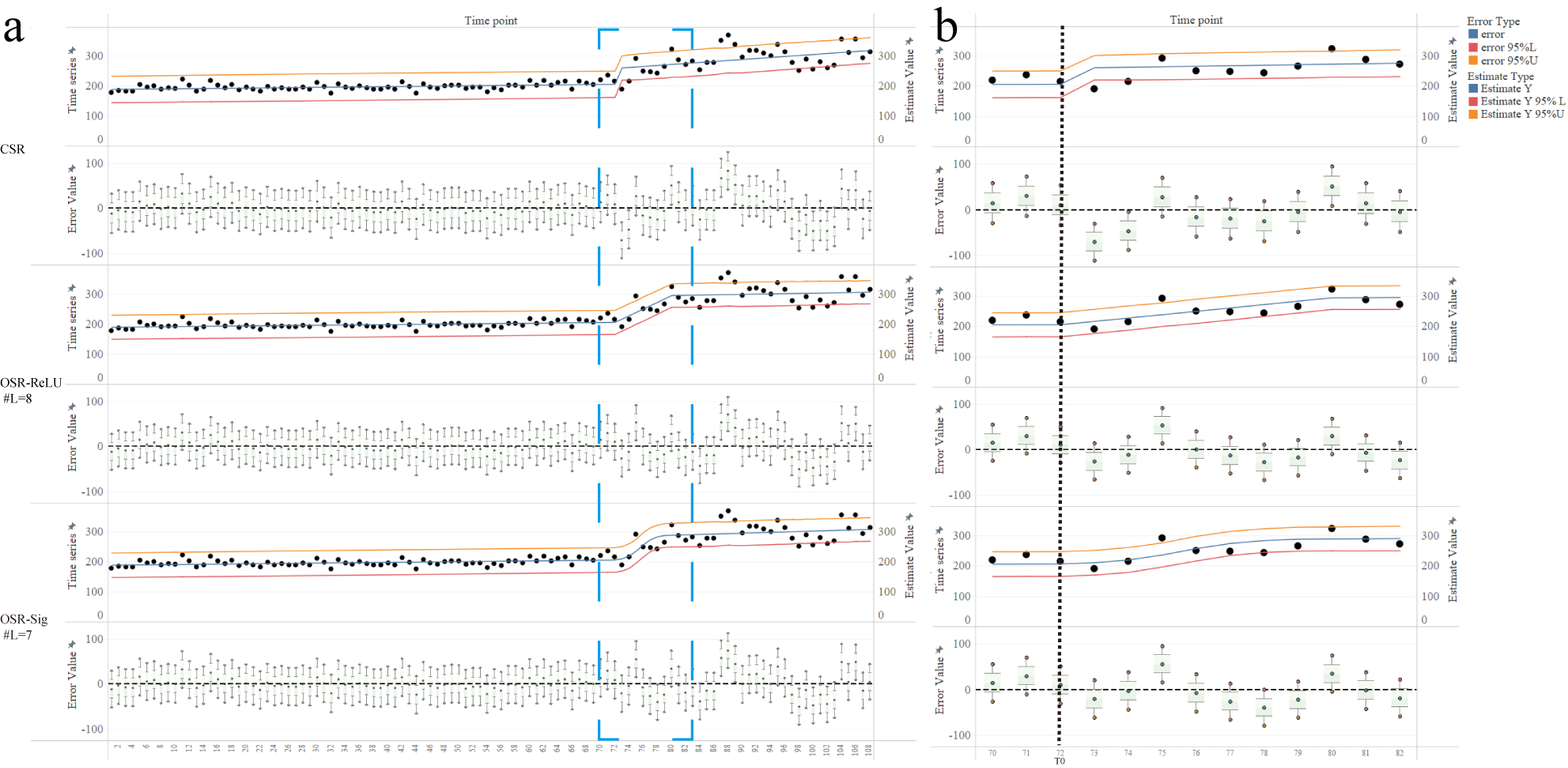


**Fig S9. Fitting curves and fitting errors of different models.**

(a) This panel shows the fitted curves and the corresponding errors for each of the three different models (CSR, OSR-ReLU, and OSR-Sig). The solid black dots indicate the values of the time series at different time points. The blue curve (Estimate Y) indicates the fitted value of the model, the red curve (Estimate Y 95%L) indicates the lower limit of the 95% confidence interval of the fitted value, and the orange curve (Estimate Y 95%U) indicates the upper limit of the 95% confidence interval of the fitted value. The box-and-whisker plot in the lower half shows the errors between the fitted value of the model and actual values of the time series, and their 95% upper and lower limits (error 95%L and error 95%U). (b) This panel is an enlarged view of the part labeled by the blue dashed square in Panel a), i.e., the fitted CSR and OSR curves and their fitting errors during the intervention lag period.

1. **Step: Estimates results of long-term impact with selected** $\boldsymbol{L}$

For intervention evaluation, the long-term impact $\beta_{3}$ is the most important evaluation indicator.[4] With the selected lag length $L$ ($L$=8 for OSR-ReLU; $L$=7 for OSR-Sig), we can estimate the long-term impact with the CSR model and OSR models (OSR-ReLU & OSR-Sig). The long-term impact estimate $\hat{\beta}_{3}$ was 1.4251 (95% CI: 0.6574, 2.1928) of the CSR model was higher than the estimates of the OSR model, specifically there were 0.1755 (95% CI: -0.6432, 0.9942) for OSR-ReLU and 0.3168 (95% CI: -0.5134, 1.1470) for OSR-Sig. Compared with the OSR models, the CSR model overestimated the long-term impact $\hat{\beta}_{3}$. If we consider further that the MSE of OSR-ReLU is smaller, we prefer to trust the estimate of OSR-ReLU: 0.1755 (95% CI: -0.6432, 0.9942).

- **Baseline simulation.**

To demonstrate the potential loss in efficiency of OSR models, we additionally designed baseline simulation scenarios when there is no activation, and satisfying basic assumptions of the CSR model (ITS design) shown (**Fig S10)**. In the baseline simulation scenarios, the estimation performance of OSR models is indeed inferior to that of the CSR model. The longer the lag length *L* set in the OSR model, the greater the error of parameter estimation and the greater the potential efficiency loss, which is in line with our expectations and logic.

Under the baseline simulation scenarios, there are no differences between linear and nonlinear lag patterns since lag length *L*=0. To maintain the same structure of the simulation scenarios as in the original manuscript, we repeatedly generated five sets of time series according to the same simulation logic and, finally, we got 30 ($2\times3\times5=30$: positive (+2) or negative (-2) long-term impact; three different $\sigma$ of white noises; five sets repetitions) different baseline simulation scenarios. Each baseline simulation scenario was repeated 1000 times, and each generated time series was used for the estimation of long-term effects by the three models respectively.


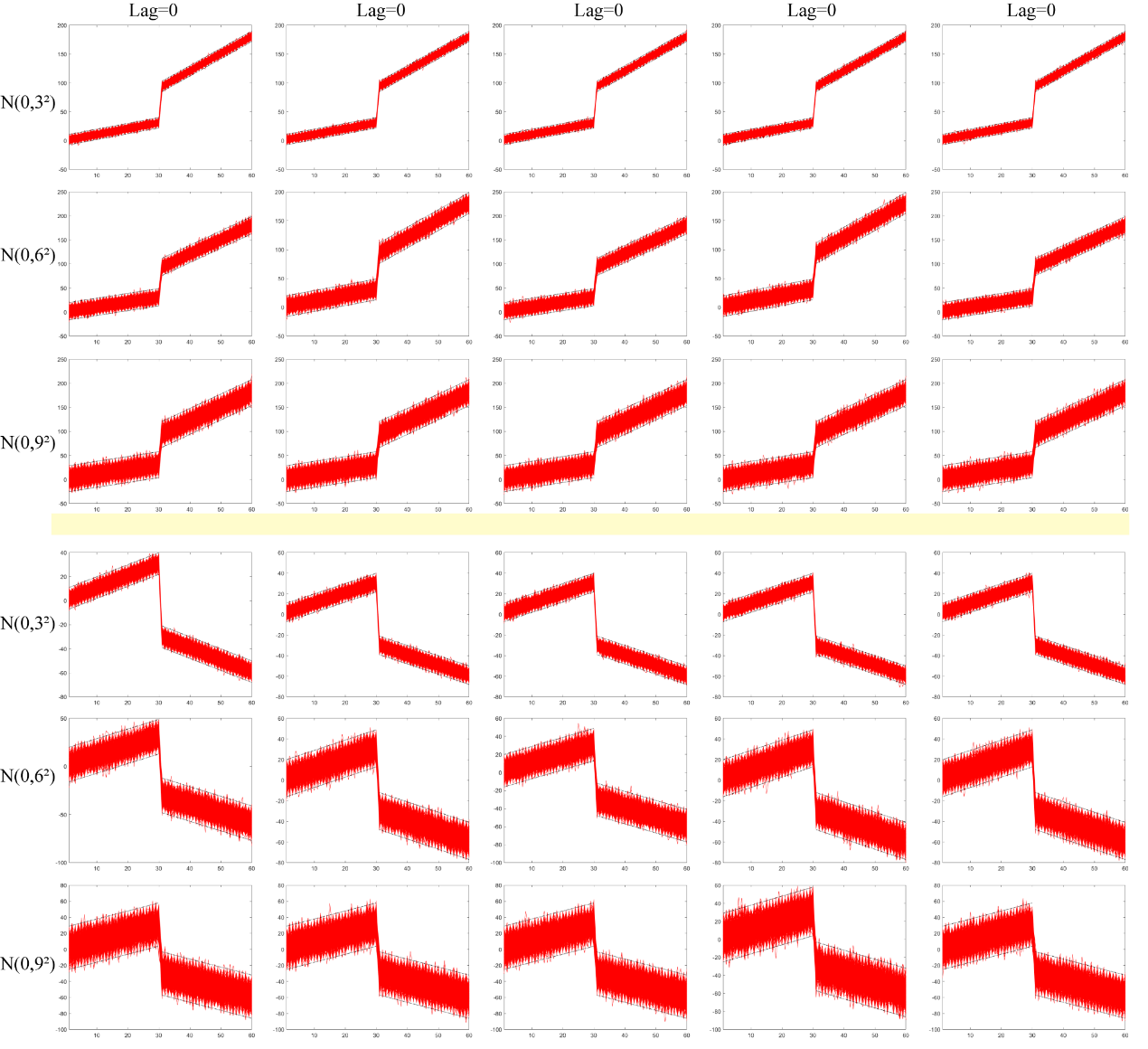


**Fig S10. Outcome time series of baseline simulation scenarios.**

The top half of the figure represents the scenarios where the simulating intervention effect is positive (+2) for the time series and the bottom half of the figure represents the scenarios where the simulating intervention is negative (-2) for the outcome time series. For one specific characteristic scenario, we repeat the simulation 1,000 times, and the specific panel shows 1,000 outcome time series.

In baseline simulation scenarios, we also evaluated the performance of different models by mean relative error, mean squared error, mean width of 95% CI, and coverage of 95% CI, see **Table S5**. In the baseline simulation scenario, the longer the lag length *L* set in the OSR model, the greater the error of parameter estimation and the greater the potential efficiency loss for OSR-RuLU and OSR-Sig (larger mean relative error, mean squared error, mean width of 95% CI, and smaller coverage of 95% CI).

**Table S5. Performance of three models in baseline simulation scenarios.**

| **Index** | **Long-term impact** | **Models** | **White noise** | **Lag length** | | | | | |
| --- | --- | --- | --- | --- | --- | --- | --- | --- | --- |
|  |  |  |  | **2** | **4** | **6** | **8** | **10** | **Mean** |
| MRE (%) | N2 | CSR | $N\left( 0,3^{2} \right)$ | 3.52 | 3.58 | 3.53 | 3.52 | 3.53 | 3.54 |
|  |  |  | $N\left( 0,6^{2} \right)$ | 6.93 | 7.16 | 6.95 | 7.00 | 7.29 | 7.07 |
|  |  |  | $N\left( 0,9^{2} \right)$ | 10.86 | 10.52 | 10.42 | 10.41 | 10.56 | 10.55 |
|  |  | OSR-ReLU | $N\left( 0,3^{2} \right)$ | 10.19 | 30.30 | 50.09 | 69.00 | 87.98 | 49.51 |
|  |  |  | $N\left( 0,6^{2} \right)$ | 11.53 | 31.14 | 49.77 | 68.81 | 87.24 | 49.70 |
|  |  |  | $N\left( 0,9^{2} \right)$ | 13.54 | 31.00 | 50.19 | 68.79 | 87.29 | 50.16 |
|  |  | OSR-Sig | $N\left( 0,3^{2} \right)$ | 10.19 | 29.87 | 47.87 | 62.98 | 75.91 | 45.36 |
|  |  |  | $N\left( 0,6^{2} \right)$ | 11.53 | 30.72 | 47.55 | 62.78 | 75.14 | 45.54 |
|  |  |  | $N\left( 0,9^{2} \right)$ | 13.54 | 30.58 | 47.97 | 62.78 | 75.25 | 46.02 |
|  | P2 | CSR | $N\left( 0,3^{2} \right)$ | 3.49 | 3.57 | 3.62 | 3.59 | 3.68 | 3.59 |
|  |  |  | $N\left( 0,6^{2} \right)$ | 7.25 | 6.95 | 7.70 | 7.25 | 7.28 | 7.29 |
|  |  |  | $N\left( 0,9^{2} \right)$ | 11.07 | 10.78 | 10.69 | 11.01 | 10.91 | 10.89 |
|  |  | OSR-ReLU | $N\left( 0,3^{2} \right)$ | 10.53 | 30.55 | 50.11 | 69.21 | 88.02 | 49.68 |
|  |  |  | $N\left( 0,6^{2} \right)$ | 11.42 | 30.39 | 49.99 | 68.82 | 87.75 | 49.67 |
|  |  |  | $N\left( 0,9^{2} \right)$ | 13.97 | 30.27 | 50.16 | 68.13 | 87.53 | 50.01 |
|  |  | OSR-Sig | $N\left( 0,3^{2} \right)$ | 10.53 | 30.13 | 47.90 | 63.19 | 75.95 | 45.54 |
|  |  |  | $N\left( 0,6^{2} \right)$ | 11.42 | 29.97 | 47.78 | 62.79 | 75.67 | 45.53 |
|  |  |  | $N\left( 0,9^{2} \right)$ | 13.97 | 29.86 | 47.94 | 62.12 | 75.44 | 45.87 |
| MSE | N2 | CSR | $N\left( 0,3^{2} \right)$ | 0.0080 | 0.0080 | 0.0080 | 0.0080 | 0.0080 | 0.0080 |
|  |  |  | $N\left( 0,6^{2} \right)$ | 0.0310 | 0.0320 | 0.0310 | 0.0310 | 0.0330 | 0.0316 |
|  |  |  | $N\left( 0,9^{2} \right)$ | 0.0750 | 0.0680 | 0.0690 | 0.0700 | 0.0710 | 0.0706 |
|  |  | OSR-ReLU | $N\left( 0,3^{2} \right)$ | 0.0490 | 0.3750 | 1.0110 | 1.9130 | 3.1050 | 1.2906 |
|  |  |  | $N\left( 0,6^{2} \right)$ | 0.0750 | 0.4210 | 1.0220 | 1.9270 | 3.0830 | 1.3056 |
|  |  |  | $N\left( 0,9^{2} \right)$ | 0.1150 | 0.4520 | 1.0800 | 1.9640 | 3.1290 | 1.3480 |
|  |  | OSR-Sig | $N\left( 0,3^{2} \right)$ | 0.0490 | 0.3650 | 0.9240 | 1.5950 | 2.3140 | 1.0494 |
|  |  |  | $N\left( 0,6^{2} \right)$ | 0.0750 | 0.4110 | 0.9360 | 1.6110 | 2.2980 | 1.0662 |
|  |  |  | $N\left( 0,9^{2} \right)$ | 0.1150 | 0.4420 | 0.9930 | 1.6490 | 2.3480 | 1.1094 |
|  | P2 | CSR | $N\left( 0,3^{2} \right)$ | 0.0080 | 0.0080 | 0.0090 | 0.0080 | 0.0080 | 0.0082 |
|  |  |  | $N\left( 0,6^{2} \right)$ | 0.0320 | 0.0310 | 0.0360 | 0.0330 | 0.0340 | 0.0332 |
|  |  |  | $N\left( 0,9^{2} \right)$ | 0.0740 | 0.0740 | 0.0720 | 0.0750 | 0.0720 | 0.0734 |
|  |  | OSR-ReLU | $N\left( 0,3^{2} \right)$ | 0.0520 | 0.3820 | 1.0130 | 1.9240 | 3.1090 | 1.2960 |
|  |  |  | $N\left( 0,6^{2} \right)$ | 0.0740 | 0.4000 | 1.0370 | 1.9290 | 3.1170 | 1.3114 |
|  |  |  | $N\left( 0,9^{2} \right)$ | 0.1170 | 0.4360 | 1.0790 | 1.9330 | 3.1520 | 1.3434 |
|  |  | OSR-Sig | $N\left( 0,3^{2} \right)$ | 0.0520 | 0.3710 | 0.9260 | 1.6060 | 2.3170 | 1.0544 |
|  |  |  | $N\left( 0,6^{2} \right)$ | 0.0740 | 0.3890 | 0.9510 | 1.6120 | 2.3280 | 1.0708 |
|  |  |  | $N\left( 0,9^{2} \right)$ | 0.1170 | 0.4260 | 0.9930 | 1.6210 | 2.3650 | 1.1044 |
| MW | N2 | CSR | $N\left( 0,3^{2} \right)$ | 0.3590 | 0.3570 | 0.3580 | 0.3560 | 0.3580 | 0.3576 |
|  |  |  | $N\left( 0,6^{2} \right)$ | 0.7110 | 0.7140 | 0.7140 | 0.7170 | 0.7110 | 0.7134 |
|  |  |  | $N\left( 0,9^{2} \right)$ | 1.0690 | 1.0690 | 1.0710 | 1.0680 | 1.0710 | 1.0696 |
|  |  | OSR-ReLU | $N\left( 0,3^{2} \right)$ | 0.5960 | 0.9320 | 1.1540 | 1.3300 | 1.4800 | 1.0984 |
|  |  |  | $N\left( 0,6^{2} \right)$ | 0.8570 | 1.1180 | 1.3160 | 1.4770 | 1.6180 | 1.2772 |
|  |  |  | $N\left( 0,9^{2} \right)$ | 1.1720 | 1.3760 | 1.5440 | 1.6860 | 1.8300 | 1.5216 |
|  |  | OSR-Sig | $N\left( 0,3^{2} \right)$ | 0.5960 | 0.9600 | 1.2330 | 1.4700 | 1.6860 | 1.1890 |
|  |  |  | $N\left( 0,6^{2} \right)$ | 0.8570 | 1.1420 | 1.3860 | 1.6060 | 1.8100 | 1.3602 |
|  |  |  | $N\left( 0,9^{2} \right)$ | 1.1720 | 1.3960 | 1.6050 | 1.8020 | 2.0020 | 1.5954 |
|  | P2 | CSR | $N\left( 0,3^{2} \right)$ | 0.3570 | 0.3560 | 0.3570 | 0.3570 | 0.3560 | 0.3566 |
|  |  |  | $N\left( 0,6^{2} \right)$ | 0.7130 | 0.7140 | 0.7150 | 0.7160 | 0.7170 | 0.7150 |
|  |  |  | $N\left( 0,9^{2} \right)$ | 1.0690 | 1.0720 | 1.0770 | 1.0710 | 1.0710 | 1.0720 |
|  |  | OSR-ReLU | $N\left( 0,3^{2} \right)$ | 0.5950 | 0.9290 | 1.1570 | 1.3300 | 1.4810 | 1.0984 |
|  |  |  | $N\left( 0,6^{2} \right)$ | 0.8590 | 1.1170 | 1.3130 | 1.4780 | 1.6190 | 1.2772 |
|  |  |  | $N\left( 0,9^{2} \right)$ | 1.1680 | 1.3760 | 1.5520 | 1.6850 | 1.8270 | 1.5216 |
|  |  | OSR-Sig | $N\left( 0,3^{2} \right)$ | 0.5950 | 0.9580 | 1.2360 | 1.4700 | 1.6860 | 1.1890 |
|  |  |  | $N\left( 0,6^{2} \right)$ | 0.8590 | 1.1410 | 1.3840 | 1.6060 | 1.8110 | 1.3602 |
|  |  |  | $N\left( 0,9^{2} \right)$ | 1.1680 | 1.3960 | 1.6130 | 1.8000 | 2.0020 | 1.5958 |
| CR (%) | N2 | CSR | $N\left( 0,3^{2} \right)$ | 95.50 | 95.40 | 96.00 | 95.00 | 93.80 | 95.14 |
|  |  |  | $N\left( 0,6^{2} \right)$ | 95.00 | 95.30 | 95.80 | 95.40 | 94.30 | 95.16 |
|  |  |  | $N\left( 0,9^{2} \right)$ | 95.40 | 96.30 | 95.40 | 95.30 | 95.30 | 95.54 |
|  |  | OSR-ReLU | $N\left( 0,3^{2} \right)$ | 84.30 | 7.00 | 0.00 | 0.00 | 0.00 | 18.26 |
|  |  |  | $N\left( 0,6^{2} \right)$ | 89.30 | 36.70 | 3.50 | 0.00 | 0.00 | 25.90 |
|  |  |  | $N\left( 0,9^{2} \right)$ | 91.20 | 59.40 | 20.30 | 2.60 | 0.20 | 34.74 |
|  |  | OSR-Sig | $N\left( 0,3^{2} \right)$ | 84.30 | 10.20 | 0.00 | 0.00 | 0.00 | 18.90 |
|  |  |  | $N\left( 0,6^{2} \right)$ | 89.30 | 41.00 | 8.60 | 0.70 | 0.10 | 27.94 |
|  |  |  | $N\left( 0,9^{2} \right)$ | 91.20 | 62.10 | 29.00 | 10.90 | 5.00 | 39.64 |
|  | P2 | CSR | $N\left( 0,3^{2} \right)$ | 95.50 | 94.40 | 93.40 | 96.40 | 94.90 | 94.92 |
|  |  |  | $N\left( 0,6^{2} \right)$ | 94.80 | 95.50 | 93.70 | 95.60 | 95.10 | 94.94 |
|  |  |  | $N\left( 0,9^{2} \right)$ | 95.60 | 94.80 | 95.20 | 95.00 | 95.80 | 95.28 |
|  |  | OSR-ReLU | $N\left( 0,3^{2} \right)$ | 82.00 | 7.10 | 0.00 | 0.00 | 0.00 | 17.82 |
|  |  |  | $N\left( 0,6^{2} \right)$ | 89.90 | 39.10 | 4.50 | 0.10 | 0.00 | 26.72 |
|  |  |  | $N\left( 0,9^{2} \right)$ | 91.60 | 63.20 | 20.30 | 2.90 | 0.30 | 35.66 |
|  |  | OSR-Sig | $N\left( 0,3^{2} \right)$ | 82.00 | 10.10 | 0.00 | 0.00 | 0.00 | 18.42 |
|  |  |  | $N\left( 0,6^{2} \right)$ | 89.90 | 42.80 | 9.70 | 0.50 | 0.10 | 28.60 |
|  |  |  | $N\left( 0,9^{2} \right)$ | 91.60 | 65.50 | 29.30 | 11.70 | 4.90 | 40.60 |

N2: negative (-2) long-term impact; P2: positive (+2) long-term impact. MRE (%): Mean relative error; MSE: Mean square error; MW: Mean width of 95% CI; CR (%): Coverage rate of 95% CI.

**Reference**

1. Demissie A, Worku A, Berhane Y. Effect of Implementing a Free Delivery Service Policy on Women’s Utilization of Facility-Based Delivery in Central Ethiopia: An Interrupted Time Series Analysis. J Pregnancy. 2020;2020:1–7.

2. Sutherland WJ, Burgman M. Policy advice: Use experts wisely. Nature. 2015;526:317–8.

3. Murphy JM, Sexton DMH, Barnett DN, Jones GS, Webb MJ, Collins M, et al. Quantification of modelling uncertainties in a large ensemble of climate change simulations. Nature. 2004;430:768–72.

4. Saldana L. The stages of implementation completion for evidence-based practice: protocol for a mixed methods study. Implementation Science. 2014;9:43.
